# Global, Regional, and National Burden of Stroke Attributable to High Systolic Blood Pressure in Adults Aged 45 Years and Above From 1990 to 2021, with Projections to 2045: A Systematic and Comprehensive Analysis of the Global Burden of Disease Study 2021

**DOI:** 10.34172/aim.34957

**Published:** 2026-02-01

**Authors:** Qian Yu, Yuan Tian, Shuang Shen, Yi Yan, Xin Liu, Zhining Liu

**Affiliations:** ^1^Huludao Central Hospital, Teaching Base of Jinzhou Medical University, Huludao, Liaoning, China; ^2^Fenyang College of Shanxi Medical University, Fenyang, China; ^3^Ultrasound Department, First Affiliated Hospital of Jinzhou Medical University, Jinzhou, Liaoning, China

**Keywords:** Global burden of disease, High systolic blood pressure, Stroke

## Abstract

**Introduction:**

Stroke remains a leading cause of death and disability worldwide, with high systolic blood pressure (HSBP) being a major modifiable risk factor. This study aimed to assess global trends, age- and SDI-specific patterns, and future projections of the stroke burden attributable to HSBP among adults aged≥45 years from 1990 to 2021.

**Methods:**

Data were obtained from the Global Burden of Disease Study 2021. Mortality, disability-adjusted life years (DALYs), age-standardized mortality rate (ASMR), and age-standardized DALYs rate (ASDR) were analyzed across sex, age, and socio-demographic index (SDI) strata. Estimated annual percentage change (EAPC) was used to quantify temporal trends, and the ARIMA model was applied for projections to 2045.

**Results:**

In 2021, global HSBP-attributable stroke deaths and DALYs among adults aged≥45 years reached 4.12 million and 86.18 million, respectively. From 1990 to 2021, the number of deaths increased by 51.5% and DALYs by 47.9%, despite declines in ASMR (EAPC=−1.72) and ASDR (EAPC=−1.61). Mortality and DALY rates increased with age in all SDI regions, peaking at 70–79 years, with low-SDI regions showing the highest rates and an earlier peak age. High-SDI regions recorded the lowest burden and largest declines in ASMR and ASDR. Projections indicate a continued global decline in mortality rates through 2045, with a faster reduction in females.

**Conclusion:**

Although the global age-standardized burden of HSBP-attributable stroke has declined, absolute cases continue to rise, especially among older adults and in low-SDI regions, highlighting the need for targeted prevention.

## Introduction

 Stroke remains one of the leading causes of death and disability worldwide, imposing substantial health and socioeconomic burdens on individuals, families, and healthcare systems.^1^ According to the Global Burden of Disease (GBD) Study 2021, stroke accounted for more than 12 million new cases and 6.5 million deaths globally in 2019, with its impact expected to grow due to population aging and the increasing prevalence of modifiable risk factors.^2-4^ Adults aged 45 years and above represent the population most vulnerable to stroke, as the incidence and mortality rates increase sharply with advancing age.^5,6^ In this demographic, the burden is not only higher but also more likely to result in long-term disability, highlighting the urgent need for targeted prevention and management strategies.

 Among the various risk factors associated with the etiology of stroke, high systolic blood pressure (SBP) has emerged as a major modifiable determinant.^5,7^ Elevated SBP accelerates vascular damage, promotes atherosclerosis, and increases the risk of both ischemic and hemorrhagic stroke.^8^ In adults aged 45 years and above, the prevalence of high SBP is particularly pronounced due to age-related vascular changes and cumulative exposure to cardiovascular risk factors.^9,10^ Over the past three decades, this trend has shown marked regional disparities, with some low- and middle-SDI countries experiencing a rapid increase in SBP-related stroke burden among middle-aged and older adults. This global epidemic poses a significant challenge to stroke prevention and control efforts.^2,11^

 Quantifying the burden of stroke attributable to high SBP in the 45 + population across different regions, sexes, and age subgroups is essential for guiding public health policies and optimizing resource allocation.^2^ Previous studies have examined the global burden of stroke and its relationship with hypertension, but few have specifically focused on middle-aged and older adults, comprehensively assessing temporal trends from 1990 to 2021 and providing robust projections for the coming decades. The GBD 2021 dataset offers a unique opportunity to fill this gap, providing detailed and comparable estimates for causes, risk factors, and disease burden for 204 countries and territories.^12^

 This study aims to analyze the global, regional, and national burden of stroke attributable to high SBP in adults aged 45 years and above from 1990 to 2021, with projections to 2045. We specifically investigated trends in age-standardized mortality rate (ASMR) and age-standardized DALY rate (ASDR) in this age group, stratified by sex and SDI regions, and explored disparities in disease burden over time. Our findings will provide valuable evidence for policymakers and healthcare providers to develop effective, evidence-based, and age-appropriate interventions to mitigate the future burden of high SBP-related stroke among adults aged 45 years and above.

## Materials and Methods

###  Data Source

 This study used data from the GBD 2021 study, obtained from the Global Health Data Exchange (https://ghdx.healthdata.org/gbd-2021). The GBD provides standardized estimates of incidence, prevalence, mortality, and disability-adjusted life years (DALYs) for 371 diseases and 88 risk factors across 204 countries and territories. High systolic blood pressure (HSBP) is recognized as a major metabolic risk factor, with its attributable burden estimated using population attributable fractions (PAFs) derived from relative risks. All estimates are age-standardized and reported with 95% uncertainty intervals (UIs). Using the GBD Results Tool (https://vizhub.healthdata.org/gbd-results/), we selected high SBP as the risk factor and stroke as the outcome. The study period covered the years 1990 to 2021. Because this study uses GBD 2021 estimates derived from the DisMod-MR 2.1 Bayesian modeling framework, the conventional concept of ‘sample size’ does not apply. Instead, the analysis is based on population-level modeled estimates that integrate diverse data sources across 204 countries and territories.

 As all data used in the GBD study are de-identified and reported in aggregate form, the Institutional Review Board of the Teaching Base of Huludao Central Hospital, affiliated with Jinzhou Medical University, approved a waiver of informed consent for this study.

###  Statistical Analysis

 Estimated Annual Percentage Change (EAPC): To quantify temporal trends in age-standardized rates, we fitted a log-linear regression model to the natural logarithm of ASRs against calendar year. The EAPC was computed as (exp(β) − 1) × 100, where β is the regression slope. The 95% confidence interval (CI) for EAPC was derived from the standard error of β using the same transformation. Regression assumptions, including normality, homoscedasticity, and linearity, were checked using diagnostic plots and were reasonably satisfied.

 Frontier analysis: We use Data Envelopment Analysis (DEA) revealed variations in the relative efficiency of countries and regions in improving health outcomes. The constructed performance frontier represented the best achievable health improvements given the input levels. Efficiency scores indicated that some countries closely approached the frontier, demonstrating high efficiency, while others showed greater distances, reflecting lower relative efficiency in health improvement.

 Socio-demographic Index (SDI) analysis was conducted using the SDI, a composite indicator of development status encompassing income, education, and fertility, developed by GBD researchers. LOESS smoothing was applied to visualize the association between age-standardized rates and SDI, using a tricube weight function. The span parameter was selected automatically based on the data, guided by minimizing the Akaike Information Criterion (AIC) and confirmed by visual inspection.

 All GBD estimates are presented with 95% uncertainty intervals (UIs), derived from 1,000 posterior draws and defined as the 2.5th and 97.5th percentiles. All statistical analyses were performed using R version 4.3.2 (R Foundation for Statistical Computing, Vienna, Austria). A p-value < 0.05 was considered statistically significant.

## Results

###  Global Trends of Stroke Attributable to High Systolic Blood Pressure

**Figure 1:**
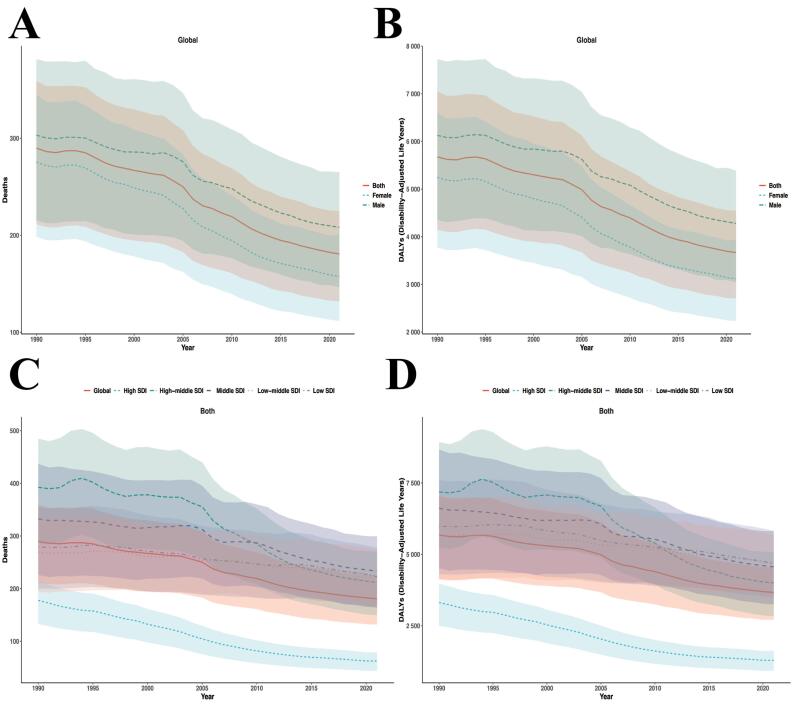
Trends in Global Age-Standardized Mortality Rate (ASMR) and Age-Standardized Disability-Adjusted Life Years Rate (ASDR) of Stroke Attributable to High Systolic Blood Pressure by Sex and SDI Level, 1990–2021. (A) ASMR by sex. (B) ASDR by sex. (C) ASMR by SDI level. (D) ASDR by SDI level. Shaded bands denote 95% uncertainty intervals (UIs) ASDR: age-standardized DALYs rate, ASMR: age-standardized mortality rate, SDI: Socio-demographic Index, DALYs: disability-adjusted life years.

 In 2021, it was estimated that stroke deaths and DALYs attributable to high systolic blood pressure (HSBP) in adults aged 45 years and above globally amounted to 4,118,353.97 (95% UI = 3,015,339.13 to 5,122,089.82) and 86,177,654.41 (95% UI = 63,747,809.91 to 106,861,988.80), respectively. Between 1990 and 2021, the number of stroke deaths attributable to HSBP in this population increased by 51.5%, rising from 2,718,488.94 (95% UI 1,988,402.04–3,365,426.93) to 4,118,353.97 (95% UI 3,015,339.13–5,122,089.82). Over the same period, HSBP-related DALYs increased by 47.9%, from 58,262,759.02 (95% UI 42,585,388.03–72,322,441.06) to 86,177,654.41 (95% UI 63,747,809.91–106,861,988.80). In 2021, the ASMR and ASDR attributable to HSBP were 180.66 (95% UI: 131.96–224.97) and 3666.02 (95% UI: 2708.53–4548.24), respectively. From 1990 to 2021, the estimated annual percentage change (EAPC) was –1.72 (95% CI: –1.84 to –1.60) for ASMR and –1.61 (95% CI: –1.73 to –1.50) for ASDR (Tables 1 and 2; Figure 1A, B).

**Table 1:** Global Burden of Stroke Deaths Attributable to High Systolic Blood Pressure among Adults Aged 45 Years and Above in 1990 and 2019, and the Estimated Annual Percentage Change (EAPC) in ASMR from 1990 to 2021

	**1990 Mortality cases** **(95% UI)**	**Mortality rate** **(95% UI)**	**2021 Mortality cases** **(95% UI)**	**Mortality rate** **(95% UI)**	**1990-2021** **EAPC (95% CI)**
Global	2718488.94(1988402.04,3365426.93)	289.73(211.38,358.92)	4118353.97(3015339.13,5122089.82)	180.66(131.96,224.97)	-1.72(-1.84,-1.60)
Sex					
Female	1478043.99(1067392.79,1847685.96)	275.51(198.49,344.72)	2015334.19(1429765.62,2569199.26)	157.45(111.79,200.65)	-2.05(-2.18,-1.92)
Male	1240444.95(880859.09,1561314.47)	303.25(215.53,381.59)	2103019.78(1484460.54,2668516.37)	208.16(146.33,264.87)	-1.36(-1.47,-1.24)
SDI					
High SDI	541638.63(407076.67,650347.96)	177.73(133.04,214.07)	422854.85(296121.50, 538871.83)	62.48(44.05,79.14)	-3.72(-3.88,-3.55)
High-middle SDI	908818.14(663704.73, 1122054.00)	392.42(285.92,484.71)	1132804.36(801001.89, 1443934.65)	211.96(149.76,270.47)	-2.37(-2.63,-2.11)
Middle SDI	750597.69(511819.30,985510.44)	332.54(225.81,436.87)	1536902.68(1084836.97, 1963668.32)	233.31(164.00,299.15)	-1.20(-1.34,-1.07)
Low-middle SDI	372586.70(268921.63,461892.94)	269.32(193.91,334.84)	770139.87(581516.42, 938834.90)	223.92(168.52,273.62)	-0.61(-0.66,-0.56)
Low SDI	140768.25(101118.85,178763.64)	278.92(199.88,354.49)	251643.82(186988.53, 313002.49)	222.68(164.69,277.71)	-0.75(-0.81,-0.69)
Regions					
Andean Latin America	4694.53(2687.20,6909.80)	93.45(53.51,137.23)	10452.43(6818.18,14635.42)	67.80(44.36,94.77)	-1.01(-1.25,-0.76)
Australasia	8727.15(6436.67,10719.49)	143.14(104.51,176.94)	7448.75(4922.55,10073.48)	43.09(28.65,58.00)	-4.13(-4.28,-3.98)
Caribbean	13016.11(9072.09,16573.34)	197.79(137.34,252.14)	20835.75(14549.79,27090.04)	139.92(97.86,181.75)	-1.08(-1.14,-1.02)
Central Asia	39557.34(28827.38,48063.89)	342.82(248.96,417.71)	51824.78(38694.22,63331.61)	281.57(209.79,344.13)	-0.99(-1.30,-0.67)
Central Europe	175751.41(135320.77,207009.47)	476.42(364.55,564.26)	132906.54(99699.73,161400.57)	201.83(151.31,245.19)	-3.18(-3.37,-2.98)
Central Latin America	24563.51(17411.10,30833.14)	128.93(91.27,162.60)	49235.92(34988.22,62222.94)	75.93(53.95,95.88)	-1.93(-2.04,-1.81)
Central Sub-Saharan Africa	16993.75(11237.56,23460.68)	365.09(242.86,502.30)	31936.56(21039.82,45454.21)	292.70(192.57,417.58)	-0.88(-0.98,-0.78)
East Asia	746307.98(434876.65, 1065770.94)	418.96(240.38,599.27)	1501096.69(942050.50,2076624.66)	275.66(172.48,382.54)	-1.43(-1.70,-1.15)
Eastern Europe	327458.56(247792.01,389151.79)	476.60(357.69,569.98)	261636.73(194381.77, 316912.44)	265.87(197.41,322.04)	-2.70(-3.19,-2.21)
Eastern Sub-Saharan Africa	48690.71(33845.78,63470.89)	291.17(201.10,379.73)	89538.59(65205.44,113377.80)	240.73(173.59,306.21)	-0.71(-0.77,-0.65)
High-income Asia Pacific	111773.29(83212.70,135205.65)	227.52(168.13,276.75)	93484.59(58728.37,126746.03)	55.41(35.87,73.61)	-4.96(-5.17,-4.75)
High-income North America	95922.64(69303.88,117597.03)	95.25(68.60,117.00)	108494.38(69003.80,146986.89)	54.46(34.83,73.48)	-2.36(-2.66,-2.05)
North Africa and Middle East	118989.58(83199.65,149916.58)	333.21(232.04,421.45)	207787.93(148178.44,261951.02)	210.21(149.30,265.34)	-1.55(-1.61,-1.48)
Oceania	1776.61(1084.76,2568.80)	289.19(177.55,414.73)	4139.98(2385.63,6190.89)	251.47(144.18,375.22)	-0.50(-0.56,-0.44)
South Asia	263853.69(188034.95,332206.27)	202.93(144.10,256.45)	586824.95(430694.61,728828.00)	164.75(120.45,205.36)	-0.76(-0.85,-0.66)
Southeast Asia	234419.11(170721.78,292707.99)	409.35(296.68,513.65)	538381.81(404886.60,659137.08)	348.54(261.45,426.27)	-0.44(-0.61,-0.28)
Southern Latin America	23310.67(15116.56,30897.51)	200.83(130.02,266.23)	21383.31(15018.03,27010.65)	85.77(60.22,108.37)	-2.41(-2.51,-2.31)
Southern Sub-Saharan Africa	13286.65(9136.84,17024.43)	213.04(145.67,274.60)	33142.92(24375.48,40793.80)	257.24(187.30,318.53)	0.75 (0.22,1.29)
Tropical Latin America	56680.77(41416.75,69356.34)	268.44(195.16,329.91)	71240.30(51755.11,87663.64)	104.89(76.13,129.21)	-2.96(-3.01,-2.90)
Western Europe	329067.27(249697.43,391900.12)	197.44(149.10,236.10)	177127.38(122529.25,222515.19)	53.81(37.54,67.23)	-4.41(-4.57,-4.26)
Western Sub-Saharan Africa	63647.60(44624.42,81761.52)	320.23(223.69,412.25)	119433.68(89859.41,148370.53)	284.88(214.13,353.32)	-0.28(-0.40,-0.15)

EAPC: estimated annual percentage change, AAPC: average annual percentage change, CI: confidence interval, GBD: Global Burden of Disease, SDI: socio-demographic index, UI: uncertainty interval.

**Table 2:** Global Burden of Stroke DALYs Attributable to High Systolic Blood Pressure among Adults Aged 45 Years and Above in 1990 and 2019, and the Estimated Annual Percentage Change (EAPC) in ASDR from 1990 to 2021

	**1990**		**2021**		**1990-2021**
	**DALYs cases(95% UI)**	**DALYs rate**	**DALYs cases**	**DALYs rate**	**EAPC (95% CI)**
Global	58262759.02(42585388.03,72322441.06)	5675.52(4147.42,7044.05)	86177654.41(63747809.91,106861988.80)	3666.02(2708.53,4548.24)	-1.61(-1.73,-1.50)
Sex					
Female	29554546.61(21301065.60,37215519.17)	5245.72(3777.10,6606.10)	39657119.21(28392814.33,50090592.37)	3119.10(2234.42,3939.30)	-1.94(-2.06,-1.81)
Male	28708212.41(20348125.89,36248314.40)	6126.86(4350.32,7726.53)	46520535.20(33166671.65,58526704.76)	4279.31(3044.47,5390.82)	-1.31(-1.42,-1.20)
SDI					
High SDI	10091227.84(7627490.92,12068210.46)	3311.08(2496.52,3968.29)	7813437.96(5588122.55,9846514.61)	1288.70(923.54,1619.54)	-3.40(-3.56,-3.23)
High-middle SDI	18374810.27(13386543.82,22845476.98)	7186.22(5230.58,8930.73)	21825733.22(15480201.27,27738215.66)	4002.76(2838.40,5091.95)	-2.29(-2.56,-2.02)
Middle SDI	17227014.13(11751942.02,22603429.99)	6614.18(4509.44,8670.64)	32681235.47(23312206.75,41565360.82)	4563.47(3249.37,5813.16)	-1.27(-1.38,-1.15)
Low-middle SDI	8982704.92(6476900.29,11113505.91)	5687.64(4102.97,7043.03)	17684827.85(13404015.53,21560139.85)	4661.81(3531.63,5685.62)	-0.67(-0.72,-0.61)
Low SDI	3505221.28(2517586.38,4460552.38)	5984.01(4301.79,7602.88)	6092700.17(4537349.68,7592223.38)	4681.46(3484.82,5830.11)	-0.86(-0.93,-0.79)
Regions					
Andean Latin America	104403.18(59588.49,154922.12)	1948.77(1115.23,2879.59)	214819.25(138033.38, 301396.66)	1353.31(873.25,1894.86)	-1.20(-1.47,-0.94)
Australasia	155903.77(116994.80,190324.03)	2464.39(1837.80,3018.96)	126967.20(86114.26, 167590.49)	802.59(544.51,1056.16)	-3.87(-4.06,-3.69)
Caribbean	281018.02(195063.85,359461.68)	4061.65(2816.10,5195.68)	441956.71(312139.32, 574562.70)	2976.65(2103.68,3868.62)	-0.96(-1.04,-0.89)
Central Asia	887210.51(648985.62, 1074794.56)	7167.12(5232.74,8691.23)	1165611.50(875264.52,1426938.77)	5586.98(4192.81,6830.86)	-1.21(-1.55,-0.87)
Central Europe	3391729.99(2635783.63, 3981502.63)	8584.72(6643.60,10108.40)	2309057.60(1753032.84,2780700.60)	3646.93(2767.47,4394.19)	-3.21(-3.41,-3.00)
Central Latin America	529602.59(375355.80,662544.47)	2511.90(1783.19,3146.74)	1028791.52(735678.76,1295567.52)	1527.09(1092.70,1921.25)	-1.88(-2.00,-1.75)
Central Sub-Saharan Africa	434083.61(287116.39,599390.58)	7709.04(5143.84,10583.46)	789758.02(522439.04, 1115738.21)	5959.30(3959.69,8396.65)	-1.00(-1.10,-0.90)
East Asia	16694780.12(9590216.80,24053168.15)	7872.23(4513.83,11310.40)	30092915.46(18784840.95,41465184.65)	5106.61(3186.33,7046.70)	-1.48(-1.72,-1.24)
Eastern Europe	6477121.43(4951408.11,7661366.60)	8732.27(6640.60,10368.62)	4902684.21(3678659.13,5904954.97)	5041.67(3780.33,6073.34)	-2.60(-3.10,-2.09)
Eastern Sub- Saharan Africa	1219967.43(849706.42,1597175.27)	6314.57(4397.83,8241.36)	2208465.41(1616940.35,2795200.60)	5143.31(3755.17,6509.98)	-0.78(-0.84,-0.72)
High-income Asia Pacific	2307936.26(1731294.17,2781011.73)	4348.15(3249.83,5250.82)	1723801.15(1148083.75,2280582.19)	1266.06(856.60,1654.59)	-4.39(-4.60,-4.19)
High-income North America	1815432.18(1332704.09,2225200.96)	1851.94(1355.34,2274.59)	2047383.85(1336939.48, 2745965.59)	1101.88(718.27,1476.62)	-2.20(-2.49,-1.90)
North Africa and Middle East	2645919.15(1854594.18,3322488.43)	6377.93(4467.17,8017.97)	4473589.20(3224212.55, 5613476.04)	3952.02(2845.86,4955.46)	-1.65(-1.70,-1.59)
Oceania	45928.72(27980.12,66558.99)	6174.98(3792.75,8872.72)	109122.94(63440.78, 162921.64)	5517.82(3211.62,8197.38)	-0.38(-0.45,-0.30)
South Asia	6560623.84(4691506.40,8244228.01)	4383.20(3130.16,5512.21)	13571408.42(9999005.80, 16830488.73)	3459.52(2545.26,4295.45)	-0.85(-0.93,-0.77)
Southeast Asia	5624884.82(4125501.39,6989442.16)	8578.61(6289.42,10680.12)	12559890.28(9487869.85, 15382268.67)	7206.78(5445.90,8804.76)	-0.50(-0.63,-0.36)
Southern Latin America	481176.47(310941.18,640933.60)	3919.55(2532.90,5217.70)	420829.05(298465.79, 532527.88)	1736.11(1229.29,2199.48)	-2.39(-2.46,-2.32)
Southern Sub- Saharan Africa	312976.72(217904.56,395905.43)	4519.71(3141.82,5736.08)	757820.58(560503.61, 933873.19)	5172.60(3813.92,6384.52)	0.57 (0.06,1.08)
Tropical Latin America	1296076.42(946124.37,1586422.88)	5451.72(3979.49,6680.16)	1511412.96(1107622.23, 1845956.80)	2160.29(1581.86,2640.54)	-3.03(-3.08,-2.97)
Western Europe	5505607.97(4219101.43,6523172.96)	3334.19(2548.55,3960.24)	2899798.27(2076360.52,3607332.36)	1007.65(726.28,1249.75)	-4.09(-4.25,-3.93)
Western Sub- Saharan Africa	1490375.84(1049280.39,1909083.43)	6615.23(4654.04,8475.40)	2821570.84(2125367.85,3510476.84)	5745.15(4340.18,7120.08)	-0.37(-0.49,-0.24)

EAPC: estimated annual percentage change, AAPC: average annual percentage change, CI: confidence interval, GBD: Global Burden of Disease, SDI: socio-demographic index, UI: uncertainty interval.

###  Geographical Variations in Stroke Burden Attributable to High Systolic Blood Pressure

 In 2021, regions with middle SDI reported the highest number of deaths (1,536,902.68; 95% UI = 1,084,836.97 to 1,963,668.32) and DALYs (32,681,235.47; 95% UI = 23,312,206.75 to 41,565,360.82) due to high systolic blood pressure in adults aged 45 years and above. In contrast, regions with low SDI reported the lowest figures for both stroke-related deaths (251,643.82; 95% UI = 186,988.53 to 313,002.49) and DALYs (6,092,700.17; 95% UI = 4,537,349.68 to 7,592,223.38). Furthermore, middle SDI regions exhibited the highest ASMR (233.31; 95% CI = 164.00 to 299.15), while low SDI regions recorded the highest ASDR (4681.46; 95% CI = 3484.82 to 5830.11) (Tables 1 and 2). From 1990 to 2021, high SDI regions experienced the largest decline in ASMR (64.8%), decreasing from 177.73 (95% UI = 133.04 to 214.07) to 62.48 (95% UI = 44.05 to 79.14), reflecting their well-developed prevention and control systems. These regions also showed the greatest reduction in ASDR, from 3311.08 (95% UI = 2496.52 to 3968.29) in 1990 to 1288.70 (95% UI = 923.54 to 1619.54) in 2021 (Figure 1C, D).

 Geographically, East Asia had the highest number of stroke-related deaths (1,501,096.69; 95% UI = 942,050.50 to 2,076,624.66) and DALYs (30,092,915.46; 95% UI = 18,784,840.95 to 41,465,184.65) in 2021 linked to high systolic blood pressure. Conversely, Australasia recorded the lowest, while Southeast Asia had the highest ASMR and ASDR. Between 1990 and 2021, the High-income Asia Pacific region experienced the largest reductions in ASMR and ASDR, whereas Southern Sub-Saharan Africa saw the most significant increases, with changes of -4.96% (95% CI = -5.17 to -4.75%) vs. 0.75% (95% CI = 0.22 to 1.29%) and -4.39% (95% CI = -4.60 to -4.19%) vs. 0.57% (95% CI = 0.06 to 1.08%), respectively (Tables 1 and 2).

###  Country-level Burden of Stroke Attributable to High Systolic Blood Pressure

**Figure 2:**
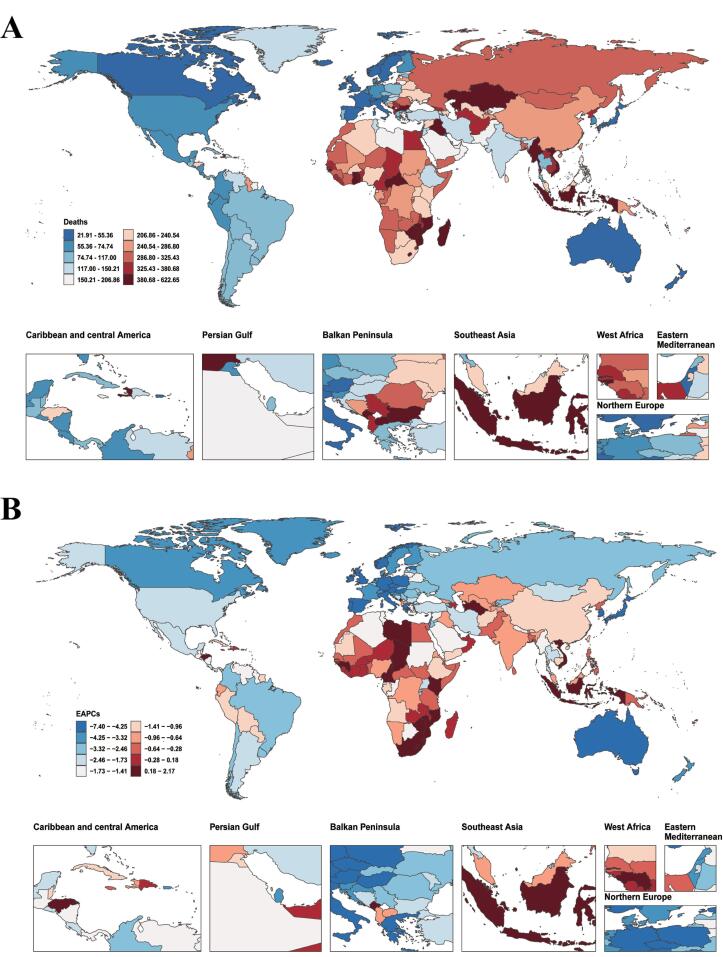
Age-standardized mortality rate (ASMR) (A) and estimated annual percentage change (EAPC) (B) of stroke attributable to high systolic blood pressure in adults aged 45 years and above across 204 countries and territories

**Figure 3:**
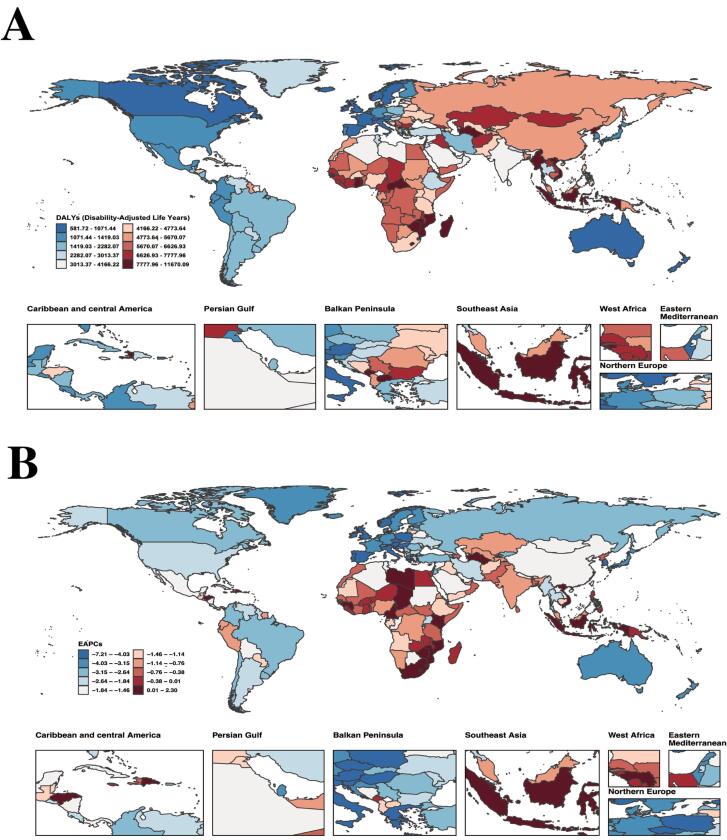
Age-standardized DALYs rate (ASDR) (A) and estimated annual percentage change (EAPC) (B) of stroke attributable to high systolic blood pressure in adults aged 45 years and above across 204 countries and territories. DALYs: disability-adjusted life years

 In 2021, at the national level among adults aged 45 years and above, China reported the highest number of stroke-related deaths and DALYs attributable to high systolic blood pressure, with 1.46 million (95% UI = 0.91 to 2.03 million) deaths and 29.22 million (95% UI = 18.09 to 40.51 million) DALYs, respectively. India followed, with 424,000 (95% UI = 307,000 to 532,000) deaths and 9.96 million (95% UI = 7.26 to 12.47 million) DALYs. The Russian Federation ranked third, reporting 190,000 (95% UI = 140,000 to 230,000) deaths and 3.51 million (95% UI = 2.62 to 4.21 million) DALYs. In the same year, among adults aged 45 years and above, Macedonia and Nauru recorded the highest ASMR and ASDR of stroke attributable to high systolic blood pressure, whereas Singapore reported the lowest values for both indicators. The countries with the largest increases in ASMR were Lesotho, Zimbabwe, and Mozambique, with EAPCs of 2.15% (95% CI = 2.15 to 2.15%), 2.07% (95% CI = 2.07 to 2.07%), and 1.14% (95% CI = 1.14 to 1.14%), respectively. Similarly, Lesotho, Zimbabwe, and Cameroon exhibited the greatest increases in ASDR, with EAPCs of 2.28% (95% CI = 2.28 to 2.28%), 2.21% (95% CI = 2.21 to 2.21%), and 0.86% (95% CI = 0.86 to 0.86%), respectively. Meanwhile, Singapore showed the most significant declines in both ASMR and ASDR, with EAPCs of –6.99% (95% CI = –6.99 to –6.99%) and –6.62% (95% CI = –6.62 to –6.62%), respectively (Figures 2 and 3; Tables S1 and S2).

###  Global Stroke Burden Attributable to High Systolic Blood Pressure by Sex and Age

 Sex-based disparities continue to influence the global burden of stroke attributable to high systolic blood pressure among individuals aged 45 years and above. In 2021, the number of stroke-related deaths and DALYs in this age group was higher in males than females, with values of 2,103,019.78 (95% UI = 1,484,460.54 to 2,668,516.37) vs. 2,015,334.19 (95% UI = 1,429,765.62 to 2,569,199.26) and 46,520,535.20 (95% UI = 33,166,671.65 to 58,526,704.76) vs. 39,657,119.21 (95% UI = 28,392,814.33 to 50,090,592.37), respectively (Table 1).

 Among adults aged 45 years and above, the ASMR of stroke attributable to high systolic blood pressure was higher in males, with values of 208.16 (95% UI = 146.33 to 264.87), compared to 157.45 (95% UI = 111.79 to 200.65) females. Similarly, the ASDR was higher in males [4279.31 (95% UI = 3044.47 to 5390.82) vs. 3119.10 (95% UI = 2234.42 to 3939.30)]. From 1990 to 2021, the EAPCs in ASMR and ASDR attributable to high systolic blood pressure were −1.36 (95% CI = −1.47 to −1.24) and −1.31 (95% CI = −1.42 to −1.20) for males, while for females, the EAPCs were −2.05 (95% CI = −2.18 to −1.92) and −1.94 (95% CI = −2.06 to −1.81), respectively (Tables 1 and 2; Figure 1A, B).

**Figure 4:**
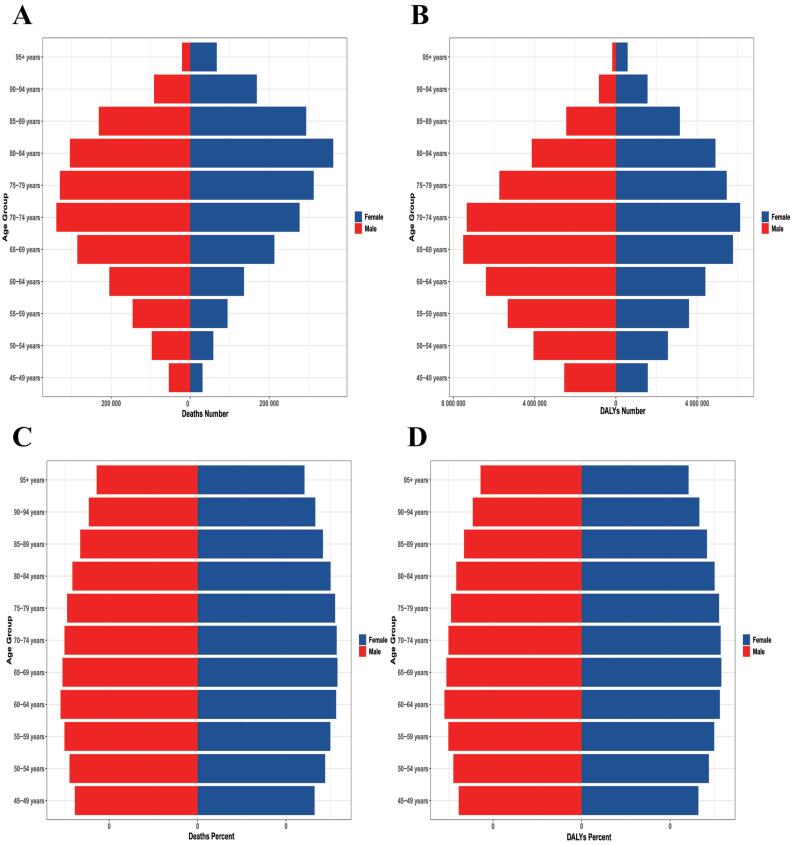
Stratification Analysis of Deaths and DALYs of Stroke Attributable to High Systolic Blood Pressure among Individuals Aged 45 Years and Above by Age and Sex in 2021; number of deaths (A), number of DALYs (B), percentage of deaths (C), and percentage of DALYs (D). DALYs, disability-adjusted life years

 When comparing male and female subgroups across different age groups, we observed that stroke deaths attributable to high systolic blood pressure increased markedly with age, peaking at 80–84 years (males: 304,524 [95% UI: 214,228–385,454]; females: 362,248 [95% UI: 253,625–452,733]). In the 45–74-year age range, the number of deaths was consistently higher in males than females. The DALY burden in males was significantly greater than females across all age groups (*P* < 0.05), displaying a clear age gradient and reaching its maximum in the 65–69-year group (males: 7,517,036 [95% UI: 5,402,197–9,420,157]; females: 5,763,075 [95% UI: 4,120,029–7,241,154]). Notably, in individuals aged ≥ 90 years, DALYs in females (578,232 [95% UI: 379,416–808,805]) exceeded those in males (179,261 [95% UI: 117,678–237,244]) (Figure 4A, B). Furthermore, the population-attributable fractions (PAFs) of stroke deaths and DALYs due to high systolic blood pressure were consistently higher in females across all age groups (Figure 4C, D).

###  Global Stroke Burden Attributable to High Systolic Blood Pressure by Age, and SDI

**Figure 5:**
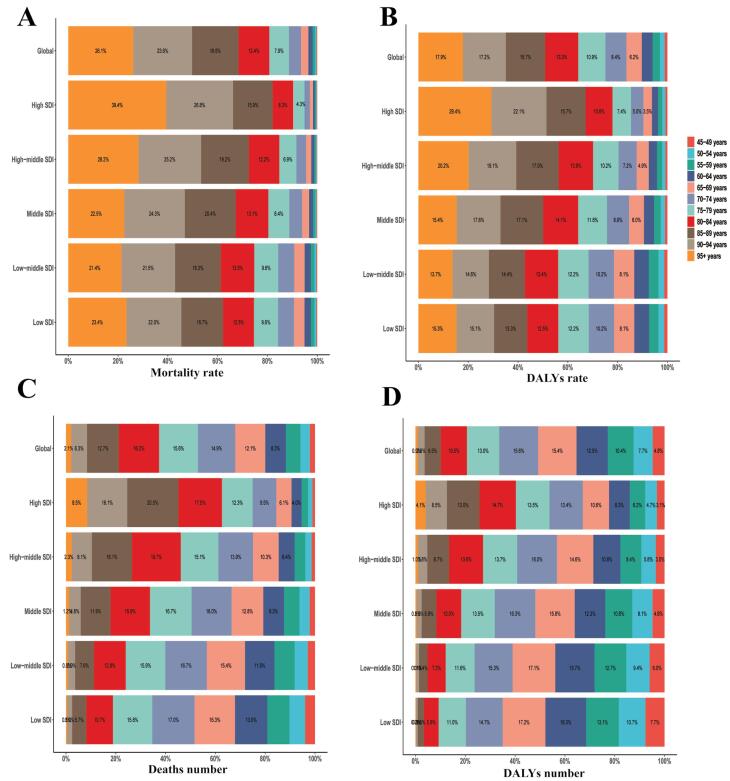
Stratification Analysis of Deaths and DALYs of Stroke Attributable to High Systolic Blood Pressure among Individuals Aged 45 Years and Above by Age and SDI Regions in 2021; mortality rate (A), DALYs rate (B), number of deaths (C), and number of DALYs (D). DALYs, disability-adjusted life years

 Among individuals aged ≥ 45 years, the mortality and DALY rates of stroke attributable to high systolic blood pressure (HSBP) increased steadily with age across all SDI regions, peaking in the 70–79-year group before declining in the oldest age groups (Figure 5A, B). Low-SDI regions consistently exhibited the highest mortality and DALY rates at each age stage, followed by middle-SDI regions, while high-SDI regions showed the lowest rates and a delayed peak age. The absolute numbers of deaths and DALYs rose sharply from midlife, reaching a maximum in the 70–79-year group in low- and middle-SDI regions, whereas in high-SDI regions, the peaks occurred later and at substantially lower counts (Figure 5C, D).

###  Factors Associated with the Burden of Stroke Attributable to High Systolic Blood Pressure

**Figure 6:**
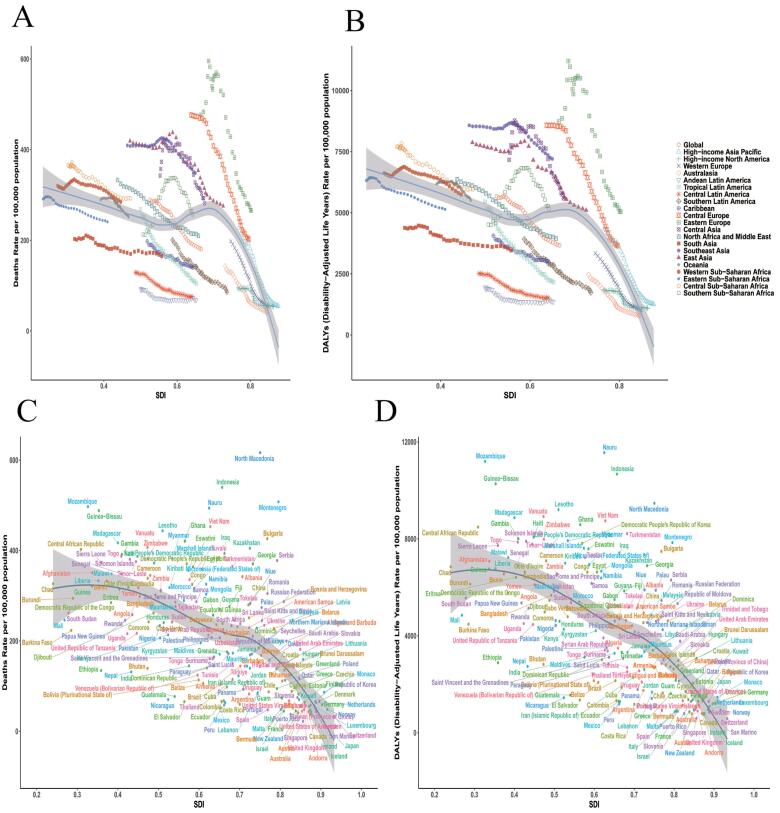
Correlation Between Stroke Attributable to High Systolic Blood Pressure in Adults Aged 45 Years and Above and SDI between 1990 and 2021; ASMR (A) and ASDR (B) in 21 GBD regions, ASMR (C) and ASDR (D) in 204 countries and territories. ASMR, age-standardized mortality rate; ASDR, age-standardized DALYs rate; GBD, Global Burden of Disease Study; DALYs, disability-adjusted life years

 A significant negative correlation was observed between ASMR and SDI (r = -0.6714, 95% CI: -0.7416 to -0.5663, *P* < 0.001). The scatterplot (Figure 6A) illustrates this inverse relationship clearly, with low-SDI regions such as Sub-Saharan Africa, Southeast Asia, and Central Asia exhibiting substantially higher ASMRs (e.g. Mozambique: 496.95/100,000; Central African Republic: 402.56/100,000), whereas high-SDI regions like Western Europe, North America, and Australasia show markedly lower rates (e.g. Switzerland: 32.75/100,000; Singapore: 22.13/100,000). This pattern highlights the important role of socioeconomic development in reducing stroke mortality through improved hypertension management. Similarly, ASDR was significantly negatively correlated with SDI (r = -0.6996, 95% CI: -0.7629 to -0.6111, *P* < 0.001) (Figure 6B). In 2021, among 204 countries and territories, the disparity between low-SDI regions (e.g. Mozambique and Central African Republic) and high-SDI regions (e.g. Switzerland and Singapore) persisted in both ASMR (Figure 6C) and ASDR. The stark contrast in ASDR between Nauru (11,554.5 per 100,000) and Singapore (599.2 per 100,000) (Figure 6D) underscores how differences in prevention and control systems significantly impact disease burden.

###  Projected Stroke Burden Attributable to High Systolic Blood Pressure

**Figure 7:**
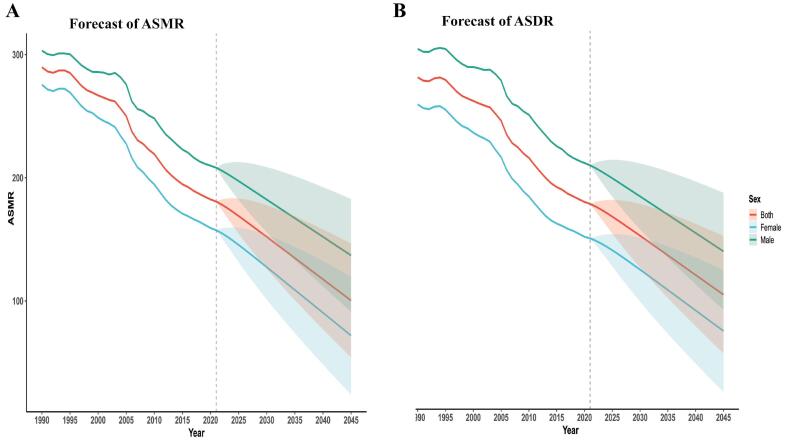
Forecasts of Age-standardized Mortality Rate (ASMR) (A) and Age-standardized Disability-adjusted Life Years Rate (ASDR) (B) of stroke attributable to high systolic blood pressure among individuals aged 45 years and above, stratified by sex, from 1990 to 2045 using the ARIMA model. ARIMA, autoregressive integrated moving average; DALYs, disability-adjusted life years

 Based on GBD estimates, the stroke burden attributable to high systolic blood pressure (HSBP) among individuals aged ≥ 45 years is projected to decline steadily from 1990 to 2045 for both sexes. Mortality rates are expected to decrease from 6126.86 to 2912.49 in males, from 5245.72 to 1647.29 in females, and from 5675.52 to 2224.10 in the total population. Historical data (1990–2021) indicate that the decline accelerated after 2005, likely reflecting the implementation of public health interventions such as vaccination programs and early screening (Figure 7A). During the projection period (2022–2045), the widening confidence intervals suggest increasing uncertainty over time, although the downward trend remains stable, with an average annual reduction of approximately 1.5% in males and 2.0% in females. The absolute mortality gap between sexes narrows over the study period (from 881.14 in 1990 to 1265.20 in 2045), with females showing a faster decline, indicating that sex-specific health policies may have contributed to the observed differences (Figure 7B).

## Discussion

###  Escalating Global Stroke Burden Attributable to High Systolic Blood Pressure and Underlying Mechanisms 

 Our study highlights a continuing global challenge posed by stroke attributable to high systolic blood pressure in adults aged 45 years and above. Between 1990 and 2021, stroke deaths and DALYs linked to HSBP increased substantially, despite declines in ASMR and ASDR. The absolute rise in stroke burden may be driven by population aging, urbanization, and the persistent prevalence of uncontrolled hypertension worldwide. These findings echo prior research emphasizing the critical role of hypertension as a major modifiable risk factor for stroke.^5,13^ The biological mechanisms linking elevated systolic blood pressure to stroke involve vascular endothelial dysfunction, increased arterial stiffness, and promotion of atherosclerosis and cerebral small vessel disease.^4,15^ Furthermore, HSBP exacerbates the risk of both ischemic and hemorrhagic strokes by elevating hemodynamic stress on cerebral vessels.^16^

###  Age and SDI Stratification Reveal Disparities in Burden and Progress

 Public health interventions, such as blood pressure management programs, have significantly reduced the burden of hypertension-related stroke, with females benefiting more than males. However, persistent sex disparities indicate a need for targeted cardiovascular health management in men. The slowdown in stroke burden decline after 2020 likely reflects the impact of the COVID-19 pandemic on chronic disease management.^17,18^

 Consistent with previous literature, our age-stratified analysis revealed a clear increase in stroke mortality and DALY rates with advancing age, peaking between 70 and 79 years before declining in the oldest age groups. The burden was disproportionately higher in low and middle SDI regions, which also exhibited earlier peak ages and substantially higher absolute mortality and DALY counts compared to high SDI regions.^19^ This disparity likely reflects differences in healthcare infrastructure, hypertension awareness, treatment coverage, and control rates across socioeconomic strata. Low-SDI regions often face challenges such as limited access to effective antihypertensive medications, inadequate health system capacity, and lower public health investment, which hinder stroke prevention efforts.^20^ In contrast, high-SDI regions benefit from better hypertension screening programs, comprehensive treatment guidelines, and public health interventions, contributing to lower stroke burden and delayed peak ages.^21^ Our findings underline the importance of socioeconomic development in shaping stroke outcomes and the critical need for equitable resource allocation to reduce global disparities.^22^

###  Sex Differences and their Implications 

 We observed that males had higher numbers of stroke deaths and DALYs attributable to HSBP compared to females across most age groups, consistent with prior epidemiological evidence. The higher male burden may be partly explained by sex-specific biological factors such as differences in vascular structure, hormonal influences, and risk factor profiles, including smoking and alcohol consumption.^23,24^ Interestingly, females showed higher population-attributable fractions of stroke burden at older ages, possibly reflecting longevity and differential risk factor accumulation.^25,26^ These patterns suggest that sex-specific prevention strategies, including tailored hypertension management and lifestyle interventions, could enhance stroke control efforts.^27^

###  Projected Trends and Public Health Implications

 Projections based on ARIMA modeling indicate a continued decline in stroke mortality rates attributable to HSBP through 2045, with a more rapid reduction observed in females. However, the expanding confidence intervals highlight increasing uncertainty over long-term forecasts, especially in low-SDI regions. The persistence of high absolute burden, particularly among aging populations, signals that stroke prevention remains a critical global health priority.^28^ Efforts to improve hypertension detection, treatment adherence, and lifestyle modification should be intensified, with particular focus on vulnerable groups in resource-limited settings. Furthermore, sex- and age-specific approaches are warranted to maximize the effectiveness of interventions.^29,30^

## Strengths and Limitations

 Our study leverages comprehensive the Global Burden of Disease data to provide a robust and nuanced assessment of stroke burden attributable to HSBP across multiple demographic and socioeconomic dimensions over three decades. The inclusion of stratified analyses by age, sex, and SDI enhances the relevance of findings for targeted public health planning. Nonetheless, several limitations merit consideration. First, the GBD data rely on modeled estimates that may be affected by data quality and completeness, especially in low-resource settings with limited vital registration systems. Second, the ARIMA model assumes stationarity and linear trends that may not fully capture future shifts in healthcare policies, medical advances, or behavioral changes. Third, residual confounding by other stroke risk factors such as diabetes, smoking, and diet is possible, as the analysis focuses specifically on high systolic blood pressure.^31,32^ Future research integrating multidimensional risk factor interactions and subnational data granularity would further refine understanding and guide precise interventions.^7^

## Policy Recommendations and Future Directions

 Our findings underscore the urgent need to strengthen global and regional stroke prevention strategies centered on improved hypertension management.^33^ Policy measures should prioritize expanding access to affordable antihypertensive medications and comprehensive blood pressure monitoring, especially in low- and middle-SDI countries.^34,35^ Public health campaigns promoting lifestyle modifications, including dietary salt reduction, physical activity, and smoking cessation, are critical components.^31,36^ Given the disproportionate burden in older adults, age-tailored screening and intervention programs are essential. Additionally, addressing socioeconomic barriers and health system inequities is fundamental to reducing stroke disparities. Future research should explore innovative community-based hypertension control models and leverage emerging technologies for remote monitoring and patient education. Strengthening data collection infrastructures in underrepresented regions will enhance surveillance and enable timely policy responses.

## Conclusion

 In summary, our study demonstrated that the global stroke burden attributable to HSBP among adults aged 45 years and above has shown a substantial increase in absolute numbers of deaths and DALYs from 1990 to 2021, despite declines in ASMR and ASDR. Regions with low and middle SDI bore the highest mortality and DALY rates, with low-SDI regions consistently exhibiting the greatest stroke burden. Age-stratified analysis indicated that stroke mortality and DALYs increased with age, peaking between 70 and 79 years, with males experiencing a higher absolute burden than females. Notably, females showed faster declines in ASMR and ASDR over time, narrowing the sex gap in stroke burden. Projections suggest a continued downward trend in mortality rates through 2045, with males and females experiencing annual reductions of approximately 1.5% and 2.0%, respectively, although the widening confidence intervals indicate growing uncertainty in long-term forecasts. These findings highlight the persistent challenge posed by HSBP-related stroke, especially in socioeconomically disadvantaged regions and aging populations. Effective hypertension control programs tailored by age, sex, and regional development level remain crucial to mitigating this burden globally. Strengthening healthcare infrastructure, improving access to antihypertensive treatment, and promoting lifestyle modifications should be prioritized, particularly in low- and middle-SDI countries, to achieve equitable reductions in stroke morbidity and mortality attributable to high systolic blood pressure.

## Supplementary Files

Supplementary file 1 contain Tables S1 and S2.
