# Supplementary material for: Global, Regional, and National Burden of Stroke Attributable to High Systolic Blood Pressure in Adults Aged 45 Years and Above From 1990 to 2021, with Projections to 2045: A Systematic and Comprehensive Analysis of the Global Burden of Disease Study 2021
Source: Arch Iran Med. 2026 Feb 1;29(2):98–111. doi: 10.34172/aim.34957 (PMC13477565; doi:10.34172/aim.34957)
Supplement: Supplementary file 1 [file aim-29-98-s001.pdf]

Arch Iran Med. February 2026;29(2):98-S1

doi:10.34172/aim.34957

http://journalaim.com

Table S1. Age-standardized Mortality Rate of Stroke Caused by High Systolic Blood Pressure among People Aged 45 and Above by Country.

**Location**	**1990**		**2021**		1990-2021
	**Mortality cases**	**Mortality rate**	**Mortality cases**	**Mortality rate**	**EAPC**
Afghanistan	7757.98(4031.01,11763.27)	474.66(245.67,718.94)	7167.65(3709.72,11138.87)	350.62(183.59,537.98)	-1.21(-1.38,-1.03)
Albania	2068.90(1458.24,2647.63)	462.68(324.69,592.03)	3887.04(2549.54,5243.37)	342.11(222.37,462.91)	-0.78(-1.00,-0.56)
Algeria	7429.51(4701.07,10565.29)	363.29(233.03,512.20)	15032.76(8886.95,22182.05)	225.13(134.13,330.02)	-1.41(-1.45,-1.36)
American Samoa	12.64(7.08,18.18)	256.40(135.99,380.76)	25.98(15.72,36.91)	229.41(136.57,327.90)	-0.59(-0.75,-0.44)
Andorra	11.69(6.72,18.00)	90.19(50.71,139.13)	20.84(10.99,32.75)	43.11(23.08,67.48)	-2.13(-2.37,-1.89)
Angola	3113.91(1788.31,4601.49)	389.71(223.32,571.97)	6842.55(4047.73,10071.53)	298.53(176.34,438.95)	-1.17(-1.28,-1.06)
Antigua and Barbuda	36.72(21.61,50.81)	234.06(136.77,324.97)	37.16(22.32,50.97)	143.26(84.42,197.97)	-1.88(-2.12,-1.63)
Argentina	15327.65(8484.26,21611.76)	188.76(103.97,265.81)	13079.75(8565.14,17166.66)	82.40(53.94,108.20)	-2.25(-2.39,-2.11)
Armenia	1568.83(938.82,2070.56)	246.46(146.11,327.25)	1709.24(1127.64,2252.26)	144.21(94.45,190.53)	-2.62(-3.03,-2.20)
Australia	7186.98(5255.40,8902.54)	142.37(103.03,177.66)	6024.73(3820.12,8324.23)	40.97(26.19,56.27)	-4.30(-4.46,-4.14)
Austria	6700.00(4259.94,8745.95)	193.18(122.32,253.70)	2764.11(1635.32,3771.03)	44.53(26.81,60.41)	-5.09(-5.47,-4.72)
Azerbaijan	3061.01(1799.42,4258.97)	258.14(148.77,361.50)	4966.95(3145.50,6834.92)	231.19(147.63,315.65)	0.04(-0.23,0.32)
Bahrain	89.14(55.39,121.65)	330.92(200.71,456.67)	195.97(119.19,277.97)	168.16(102.59,238.52)	-2.79(-3.27,-2.31)
Bangladesh	39325.85(23000.26,57113.00)	355.21(208.00,515.47)	99166.16(62773.07,139534.71)	301.33(189.79,425.18)	-0.50(-0.65,-0.35)
Barbados	183.14(126.52,236.58)	215.08(147.06,280.01)	219.89(146.41,297.85)	153.16(101.71,207.74)	-1.30(-1.47,-1.13)
Belarus	10455.12(6773.08,13398.10)	303.83(194.89,390.86)	10219.88(6793.43,13642.94)	227.06(150.92,303.32)	-1.63(-2.04,-1.21)

Belgium	7136.66(4552.82,9385.26)	162.46(102.92,214.94)	4567.64(3029.90,5972.60)	56.77(38.16,73.66)	-3.25(-3.31,-3.19)
Belize	33.34(20.16,45.04)	134.99(81.66,182.20)	75.21(46.08,103.41)	105.69(65.10,144.98)	-1.28(-1.84,-0.73)
Benin	1873.15(1087.72,2638.73)	391.66(226.20,553.45)	3713.68(2391.55,5182.23)	332.18(211.21,463.80)	-0.46(-0.56,-0.37)
Bermuda	22.49(13.28,31.23)	145.12(83.96,203.13)	25.21(15.36,35.31)	60.18(36.80,84.18)	-3.02(-3.15,-2.88)
Bhutan	105.12(54.45,167.55)	199.72(100.05,323.09)	211.06(120.85,314.64)	139.45(78.88,208.58)	-1.20(-1.28,-1.12)
Bolivia	1267.80(509.40,2243.62)	170.40(68.63,299.18)	2397.45(1118.19,4104.18)	113.61(53.91,191.70)	-1.14(-1.32,-0.95)
Bosnia and Herzegovina	3489.80(2390.58,4438.71)	391.54(264.63,500.52)	4456.94(2814.93,6010.09)	251.00(157.89,339.74)	-1.73(-1.92,-1.53)
Botswana	465.00(278.87,670.33)	397.56(232.72,580.64)	716.78(437.83,1040.96)	235.62(140.56,344.10)	-1.59(-1.85,-1.34)
Brazil	55445.65(40511.68,67921.89)	269.59(195.96,331.65)	69113.79(50211.65,85161.11)	104.01(75.49,128.32)	-3.00(-3.06,-2.94)
Brunei	66.32(41.42,91.63)	308.41(187.69,428.90)	88.50(53.04,127.06)	135.32(75.96,198.77)	-2.48(-2.77,-2.18)
Bulgaria	18766.33(13484.55,23136.60)	713.43(502.34,883.97)	17347.49(11354.04,22832.63)	426.11(274.88,563.46)	-1.87(-2.06,-1.68)
Burkina Faso	2234.10(1289.11,3349.79)	231.11(131.97,346.47)	4510.08(2693.07,6529.97)	223.30(132.93,321.70)	0.18(0.06,0.29)
Burundi	2576.24(1329.86,3988.83)	460.45(235.22,715.86)	3012.10(1767.80,4432.03)	293.97(172.93,434.32)	-1.94(-2.15,-1.72)
Cambodia	4241.64(2223.45,6340.59)	439.32(232.25,651.78)	8497.90(4507.55,12834.82)	338.46(182.32,503.57)	-0.99(-1.12,-0.87)
Cameroon	2619.28(1616.71,3821.44)	268.87(166.55,390.20)	9194.86(5824.92,13386.88)	345.08(220.58,497.76)	0.92(0.36,1.48)
Canada	8612.27(5994.71,11048.98)	99.07(68.59,127.48)	8704.32(4798.23,12380.86)	37.54(20.81,53.34)	-3.49(-3.69,-3.30)
Cape Verde	130.28(77.58,185.76)	200.64(119.31,286.68)	271.56(170.39,381.80)	241.63(151.84,338.88)	0.34(0.01,0.68)
Central African Republic	1132.03(623.00,1753.45)	474.53(264.09,728.65)	1735.48(953.06,2748.25)	402.56(219.29,625.40)	-0.61(-0.68,-0.54)
Chad	2060.61(1140.25,3063.35)	300.54(163.88,448.62)	4147.70(2456.57,6185.25)	326.29(192.06,484.98)	0.24(0.05,0.42)
Chile	5433.11(3965.80,6663.87)	222.54(161.94,273.66)	6326.08(4464.32,8024.68)	87.38(61.65,110.92)	-2.64(-2.80,-2.48)
China	720832.42(412662.96,1036812.99)	422.51(237.87,608.62)	1463006.03(911393.59,2031472.48)	279.76(173.69,389.72)	-1.40(-1.69,-1.12)
Colombia	6236.51(3953.87,8217.22)	152.70(96.47,201.26)	9989.70(6770.32,13282.01)	65.68(44.93,86.98)	-3.20(-3.36,-3.04)

Comoros	151.50(82.71,229.73)	340.79(181.76,518.27)	269.61(162.81,392.74)	242.09(145.52,351.85)	-1.37(-1.55,-1.20)
Congo	1074.84(653.29,1536.56)	467.44(280.81,668.44)	1745.61(1056.98,2529.64)	324.86(195.94,466.09)	-1.46(-1.60,-1.32)
Cook Islands	5.87(2.82,9.20)	201.43(95.15,317.60)	8.93(5.38,12.88)	126.83(75.84,183.50)	-1.60(-1.76,-1.44)
Costa Rica	454.43(313.48,581.65)	103.44(71.28,132.51)	910.03(609.12,1199.03)	59.79(40.27,78.43)	-2.31(-2.66,-1.96)
Cote d'Ivoire	2766.39(1652.93,3997.19)	339.51(201.03,488.62)	7398.53(4608.01,10659.24)	318.68(199.73,456.93)	-0.21(-0.40,-0.02)
Croatia	6863.56(4915.46,8435.02)	479.85(341.54,591.03)	4163.64(2742.63,5470.26)	149.62(98.21,197.17)	-3.97(-4.12,-3.82)
Cuba	3718.28(2335.97,5021.53)	140.42(87.66,190.39)	5627.77(3278.66,7923.82)	98.07(57.38,137.78)	-1.39(-1.52,-1.27)
Cyprus	525.01(328.58,743.24)	382.46(230.30,543.86)	442.19(255.59,637.49)	96.80(52.56,141.44)	-4.70(-5.04,-4.36)
Czech Republic	16273.03(10666.86,20789.93)	441.93(289.20,565.93)	5725.31(3568.43,7629.81)	88.62(55.29,118.05)	-5.44(-5.80,-5.08)
Democratic Republic of the Congo	11021.26(6814.60,16103.34)	344.01(214.40,501.37)	20810.28(12262.02,31996.88)	284.70(167.73,439.95)	-0.74(-0.86,-0.61)
Denmark	4242.73(3218.71,5146.14)	174.33(131.70,212.04)	2517.42(1593.61,3367.12)	66.48(42.28,88.69)	-3.48(-3.68,-3.27)
Djibouti	78.04(40.18,123.82)	289.76(149.10,457.43)	325.97(188.52,500.52)	261.60(153.71,396.97)	-0.43(-0.51,-0.36)
Dominica	39.08(22.88,54.50)	245.37(141.67,344.93)	39.32(21.75,57.47)	184.93(100.93,271.10)	-0.91(-1.04,-0.79)
Dominican Republic	1427.48(781.40,2083.40)	168.74(91.93,245.96)	3710.31(2105.80,5625.54)	140.19(79.58,212.46)	-0.01(-0.24,0.23)
Ecuador	1206.10(526.51,1916.33)	95.01(41.21,151.03)	2776.90(1474.71,4205.55)	67.62(36.13,101.91)	-0.83(-1.08,-0.57)
Egypt	22597.14(12520.87,32884.99)	460.86(252.77,673.18)	42066.40(26594.81,59228.23)	358.49(220.84,506.07)	-0.28(-0.48,-0.08)
El Salvador	874.68(501.94,1251.32)	114.15(65.78,162.93)	1171.56(676.93,1740.95)	65.40(38.09,97.03)	-1.96(-2.31,-1.61)
Equatorial Guinea	189.65(109.60,282.71)	441.78(255.86,656.80)	240.33(133.63,385.81)	238.61(134.23,377.77)	-2.39(-2.72,-2.05)
Eritrea	927.98(469.91,1461.26)	403.31(196.10,631.13)	1675.93(943.04,2541.67)	314.48(178.13,469.98)	-0.74(-0.84,-0.63)
Estonia	2176.67(1439.64,2762.53)	396.61(259.68,506.34)	719.42(472.53,951.84)	82.32(54.60,108.63)	-6.51(-7.09,-5.93)
Ethiopia	7774.30(3864.59,12782.79)	171.62(83.47,282.00)	13477.71(7762.38,19425.58)	134.44(75.01,197.02)	-1.02(-1.10,-0.93)

Federated States of Micronesia	46.97(26.46,70.02)	407.95(220.24,622.25)	51.55(26.19,80.93)	314.62(155.72,493.75)	-0.89(-0.98,-0.79)
Fiji	208.24(113.36,309.81)	266.87(138.28,404.70)	457.17(283.28,653.22)	275.73(165.66,395.44)	-0.17(-0.40,0.05)
Finland	4008.65(3042.01,4831.18)	200.07(150.09,242.93)	2753.34(1703.09,3722.56)	63.88(40.45,85.51)	-3.70(-3.88,-3.52)
France	33978.94(24082.42,43385.70)	136.91(96.63,175.44)	24127.58(13904.25,33530.11)	45.06(26.60,61.94)	-3.59(-3.71,-3.48)
Gabon	462.06(276.15,669.94)	339.59(200.77,493.93)	562.31(332.50,834.62)	256.92(151.96,379.97)	-1.03(-1.17,-0.89)
Georgia	7265.25(5038.78,9086.30)	449.44(305.90,567.50)	6566.78(4368.46,8501.04)	370.25(247.78,477.08)	-0.77(-1.07,-0.46)
Germany	79797.28(59799.83,96610.23)	213.49(158.95,259.63)	39033.65(25735.97,51098.84)	58.56(38.67,76.69)	-4.25(-4.57,-3.93)
Ghana	5972.01(3961.34,8236.17)	440.32(292.34,604.05)	14887.11(10105.72,20384.89)	420.73(286.34,573.00)	0.17(-0.04,0.39)
Greece	13854.42(10315.99,16813.52)	348.47(257.32,426.02)	8591.78(5248.91,11786.66)	94.53(58.33,128.95)	-4.98(-5.38,-4.58)
Greenland	21.39(12.19,30.25)	361.94(208.54,508.52)	17.16(9.12,26.16)	125.18(67.16,189.24)	-3.64(-3.81,-3.46)
Grenada	65.71(37.88,91.12)	305.18(174.23,424.56)	47.16(27.12,65.11)	175.68(96.22,245.59)	-1.78(-1.98,-1.57)
Guam	21.53(9.77,33.10)	146.85(62.65,231.84)	43.33(26.09,61.19)	73.56(44.61,103.41)	-1.92(-2.30,-1.55)
Guatemala	735.46(395.50,1057.74)	103.58(55.36,150.36)	2067.21(1244.99,2887.70)	76.99(45.11,108.98)	-1.50(-1.82,-1.19)
Guinea	2423.53(1387.14,3594.36)	303.32(172.35,450.44)	4184.12(2509.49,6144.81)	321.13(191.00,470.05)	0.50(0.34,0.67)
Guinea-Bissau	464.78(266.11,694.75)	514.60(294.88,761.19)	719.42(439.45,1029.14)	488.26(299.36,690.73)	0.03(-0.06,0.11)
Guyana	477.18(274.29,655.54)	517.76(297.92,710.45)	409.74(232.91,602.23)	270.06(152.33,396.09)	-1.63(-1.84,-1.41)
Haiti	3633.53(2071.33,5321.48)	505.17(282.65,745.41)	6175.37(3649.01,9086.62)	387.20(222.09,574.32)	-0.66(-0.73,-0.58)
Honduras	854.53(523.41,1195.96)	179.44(109.09,252.55)	3521.60(2117.67,5101.35)	240.54(144.07,347.42)	1.13(0.87,1.39)
Hungary	18565.16(14597.07,21666.95)	490.37(384.32,574.31)	8150.06(5657.43,10454.16)	138.07(95.72,177.32)	-4.43(-4.63,-4.23)
Iceland	98.00(63.67,130.21)	116.01(75.56,153.99)	81.67(49.45,112.83)	42.56(26.14,58.28)	-3.32(-3.52,-3.11)
India	190879.78(135766.15,242817.11)	180.97(128.29,231.09)	423901.51(307380.47,531952.61)	144.73(104.72,182.08)	-0.78(-0.88,-0.68)
Indonesia	103206.84(75674.64,128644.70)	483.61(351.15,609.61)	269473.68(199838.88,338036.52)	539.65(400.59,670.42)	0.46(0.29,0.63)
Iran	11542.72(8270.82,14321.28)	234.93(167.03,294.10)	22005.58(15701.98,27607.47)	122.68(87.11,154.69)	-2.24(-2.38,-2.10)

Iraq	8518.23(5435.56,11795.86)	431.08(275.10,596.45)	19313.86(12850.59,26268.83)	393.55(264.97,528.93)	-0.90(-1.11,-0.69)
Ireland	2077.34(1426.68,2626.05)	191.73(128.39,245.66)	1110.08(702.88,1487.45)	46.87(29.72,62.78)	-4.49(-4.71,-4.28)
Israel	1734.91(1294.48,2129.73)	136.90(100.57,170.10)	1701.35(1128.45,2238.04)	44.49(29.64,58.32)	-4.03(-4.21,-3.84)
Italy	50674.60(37492.23,61274.22)	207.42(151.56,252.98)	29537.08(17177.41,41746.15)	53.99(31.98,75.53)	-4.67(-4.84,-4.50)
Jamaica	1084.80(712.87,1432.73)	213.51(139.79,282.71)	1530.16(879.31,2204.44)	172.87(101.18,246.99)	-0.42(-0.83,-0.01)
Japan	83026.32(62522.35,99635.72)	195.64(145.93,236.39)	76288.19(46952.76,105684.13)	55.07(35.47,73.80)	-4.36(-4.55,-4.16)
Jordan	793.29(499.13,1117.73)	301.85(191.61,423.74)	1953.67(1225.64,2748.31)	139.42(87.28,195.79)	-3.05(-3.43,-2.66)
Kazakhstan	12879.61(8907.10,16293.33)	429.63(296.13,543.35)	15356.00(11138.38,19260.68)	404.99(293.48,506.37)	-0.65(-1.05,-0.25)
Kenya	3614.15(2154.02,5135.68)	198.54(117.64,281.69)	9993.10(6499.82,13654.57)	213.27(136.26,293.73)	0.51(0.36,0.67)
Kiribati	25.73(12.47,40.75)	302.44(141.79,487.28)	48.58(26.04,76.03)	317.87(171.33,494.64)	0.07(-0.03,0.18)
Kuwait	120.25(79.20,158.92)	107.17(71.61,141.03)	400.73(264.77,549.30)	70.00(46.60,95.22)	-1.25(-2.34,-0.14)
Kyrgyzstan	2917.25(1731.93,3987.18)	396.03(232.19,544.22)	2174.99(1299.82,3027.32)	195.52(119.63,268.40)	-2.78(-3.26,-2.29)
Laos	2782.72(1589.64,4145.82)	579.00(325.74,862.97)	3801.58(2254.66,5534.55)	372.25(220.00,540.01)	-1.59(-1.67,-1.51)
Latvia	4434.76(2917.88,5683.34)	456.87(298.98,587.65)	3188.71(2087.48,4199.94)	245.86(162.09,322.62)	-2.63(-2.96,-2.30)
Lebanon	1299.73(739.67,1950.08)	263.59(149.31,396.16)	1535.17(949.57,2176.56)	85.37(53.06,121.06)	-3.85(-4.12,-3.57)
Lesotho	597.24(327.02,905.82)	300.96(161.59,461.11)	1062.62(581.58,1641.37)	443.70(234.42,682.13)	2.15(1.64,2.67)
Liberia	924.45(562.36,1312.83)	347.38(210.65,493.92)	1472.67(914.75,2144.47)	332.86(208.49,483.18)	-0.23(-0.35,-0.11)
Libya	760.44(472.35,1118.79)	173.06(107.93,254.18)	1958.34(1156.17,2964.45)	168.79(99.83,254.85)	0.31(0.13,0.50)
Lithuania	2810.50(1946.45,3555.02)	227.83(157.08,288.64)	2935.28(1917.07,3824.07)	158.77(104.64,206.11)	-1.46(-1.77,-1.15)
Luxembourg	408.21(266.86,520.89)	277.47(178.55,357.48)	186.01(114.24,252.65)	55.36(34.49,74.74)	-5.06(-5.15,-4.97)
Macedonia	2970.89(2082.22,3748.67)	684.11(480.45,862.05)	4154.75(2690.49,5591.52)	616.48(389.13,836.01)	-0.66(-1.13,-0.19)
Madagascar	4920.52(2591.99,7345.80)	416.08(217.39,623.09)	9598.29(5487.68,14323.84)	416.83(237.76,615.33)	-0.00(-0.07,0.07)
Malawi	2849.53(1718.68,4038.70)	326.19(188.79,471.31)	5544.71(3613.38,7646.39)	348.25(220.46,482.99)	-0.06(-0.34,0.22)

Malaysia	6976.43(4968.15,8904.41)	311.70(222.60,397.26)	15334.66(10974.81,19880.93)	228.05(162.18,296.63)	-0.72(-0.91,-0.53)
Maldives	91.43(58.36,123.88)	459.32(281.13,633.80)	125.27(78.07,173.02)	170.81(105.96,235.76)	-3.59(-3.71,-3.46)
Mali	2415.51(1331.50,3564.56)	288.98(161.39,422.86)	4484.50(2628.31,6611.24)	246.26(147.61,357.68)	-0.33(-0.45,-0.21)
Malta	211.73(141.56,272.06)	193.93(127.86,251.04)	150.33(87.54,208.88)	48.24(28.34,66.81)	-4.53(-4.77,-4.29)
Marshall Islands	13.66(6.60,21.86)	372.85(171.58,608.19)	24.87(12.53,38.58)	356.07(186.89,542.28)	-0.15(-0.26,-0.03)
Mauritania	954.93(570.87,1374.10)	404.90(237.16,585.44)	1450.74(867.78,2176.57)	295.15(177.26,441.34)	-1.13(-1.30,-0.96)
Mauritius	631.65(463.84,786.00)	367.38(267.91,458.54)	683.44(453.97,882.78)	144.28(95.51,186.78)	-4.28(-4.87,-3.69)
Mexico	10490.66(6686.70,14046.28)	113.54(72.49,152.95)	20163.57(13403.26,26602.64)	63.46(42.13,83.92)	-1.87(-1.98,-1.76)
Moldova	4031.60(2652.84,5142.77)	397.26(255.45,512.06)	3494.80(2451.15,4411.33)	210.22(147.25,265.51)	-2.37(-2.64,-2.11)
Monaco	52.77(32.33,74.24)	227.42(138.57,322.38)	29.72(16.55,43.78)	86.24(48.65,127.02)	-3.26(-3.47,-3.05)
Mongolia	1185.36(797.39,1585.98)	468.95(314.62,628.57)	1582.76(1054.88,2136.24)	303.30(197.93,412.56)	-1.73(-2.27,-1.19)
Montenegro	698.51(479.10,900.87)	445.28(304.42,574.75)	1199.84(770.38,1618.51)	507.75(323.78,686.79)	0.39(0.11,0.67)
Morocco	11760.72(7666.16,16374.77)	349.23(226.88,485.74)	22901.24(14579.61,32256.05)	289.05(184.04,406.79)	-0.56(-0.59,-0.54)
Mozambique	5754.99(3559.76,8072.69)	402.62(238.26,577.79)	12731.05(7927.22,17915.31)	496.95(302.23,704.35)	1.14(0.92,1.35)
Myanmar	32089.50(20145.29,44753.61)	595.51(375.89,829.86)	44241.41(28601.80,62394.60)	382.45(246.10,540.38)	-1.71(-1.88,-1.54)
Namibia	542.80(320.62,771.53)	399.41(231.66,572.89)	946.14(547.64,1387.03)	325.43(183.73,478.55)	-0.92(-1.21,-0.62)
Nauru	5.46(3.56,7.72)	547.96(358.46,771.75)	6.54(4.04,9.50)	493.88(294.54,720.16)	-0.45(-0.84,-0.05)
Nepal	4493.80(2384.62,6890.89)	212.39(110.83,327.74)	7351.98(4049.51,11314.18)	131.37(71.07,203.74)	-1.52(-1.58,-1.45)
Netherlands	7840.90(5659.60,9785.62)	138.27(99.42,173.21)	7009.70(4188.91,9483.07)	63.14(37.85,85.29)	-3.35(-3.75,-2.94)
New Zealand	1540.16(1148.44,1867.56)	146.93(108.88,178.99)	1424.03(930.89,1913.05)	55.17(36.13,73.96)	-3.32(-3.46,-3.19)
Nicaragua	443.69(266.68,614.48)	123.45(73.73,171.46)	826.12(501.36,1200.29)	70.01(42.04,101.97)	-1.69(-1.89,-1.49)
Niger	1727.37(951.62,2613.66)	301.15(164.52,454.46)	5077.50(2920.58,7650.42)	296.07(171.79,442.79)	0.10(0.03,0.16)
Nigeria	30743.49(19175.11,42691.96)	303.32(189.20,421.47)	44800.43(31395.37,60073.04)	233.89(164.47,311.19)	-0.82(-0.96,-0.67)

Niue	2.21(1.25,3.25)	338.06(191.49,497.45)	1.61(1.06,2.22)	282.76(184.50,389.10)	-0.86(-0.96,-0.75)
North Korea	15381.95(8449.70,22835.25)	425.56(227.83,640.16)	31192.59(17309.35,45966.84)	370.39(202.65,549.19)	-0.44(-0.64,-0.24)
Northern Mariana Islands	7.63(3.77,12.04)	243.06(119.29,380.25)	22.26(13.36,31.49)	203.65(121.46,288.21)	-0.84(-1.06,-0.62)
Norway	3704.03(2777.89,4468.92)	173.94(130.38,209.95)	1605.06(1037.56,2115.37)	48.30(31.55,63.17)	-4.39(-4.55,-4.22)
Oman	294.50(148.56,467.17)	196.75(98.89,313.20)	561.17(352.47,804.10)	157.70(98.86,225.38)	0.09(-0.26,0.43)
Pakistan	29049.15(19010.03,39531.17)	215.38(140.28,294.08)	56194.24(36647.78,77605.29)	206.28(133.84,285.68)	-0.43(-0.64,-0.23)
Palau	6.46(3.16,10.13)	282.71(134.69,448.12)	13.40(7.90,19.95)	273.85(159.19,404.31)	-0.01(-0.11,0.10)
Palestine	694.54(418.97,995.87)	363.36(215.53,522.70)	981.70(588.74,1407.39)	207.54(122.96,300.00)	-1.89(-2.24,-1.55)
Panama	543.14(346.96,718.41)	146.02(93.12,193.28)	1154.10(700.83,1596.93)	92.95(56.65,128.14)	-1.71(-1.90,-1.51)
Papua New Guinea	1045.43(553.97,1647.43)	285.14(152.32,447.04)	2686.60(1221.39,4470.01)	245.57(104.09,415.78)	-0.47(-0.58,-0.37)
Paraguay	1235.12(810.08,1665.98)	224.22(145.64,303.63)	2126.51(1256.41,3127.93)	144.99(86.01,212.45)	-1.23(-1.39,-1.07)
Peru	2220.62(1017.96,3653.82)	74.71(34.29,122.70)	5278.08(3269.15,7823.28)	58.40(36.34,86.40)	-0.96(-1.42,-0.51)
Philippines	15071.93(9958.77,19979.48)	243.14(160.50,324.05)	40451.31(25711.49,54745.67)	206.86(131.18,280.12)	-0.38(-0.54,-0.22)
Poland	41433.31(28391.73,51931.32)	373.09(251.54,472.11)	23987.54(15393.05,32073.90)	112.99(72.94,150.69)	-4.25(-4.45,-4.05)
Portugal	16292.94(10479.63,21205.75)	468.25(292.95,617.32)	7171.00(4382.76,9809.54)	83.48(51.74,113.29)	-6.10(-6.34,-5.86)
Puerto Rico	881.71(528.04,1196.56)	96.05(56.64,131.08)	875.66(510.62,1248.80)	37.27(22.09,52.71)	-3.47(-3.70,-3.24)
Qatar	47.10(29.12,67.50)	327.92(201.61,464.74)	127.33(75.85,189.13)	117.00(71.70,168.62)	-3.94(-4.75,-3.12)
Romania	37094.12(27627.57,44405.27)	577.01(423.52,699.25)	34659.34(25231.41,43424.01)	304.69(221.43,382.56)	-2.68(-2.99,-2.37)
Russian Federation	225772.81(171790.58,266559.29)	522.96(395.48,621.13)	189769.63(140491.42,230032.70)	286.90(212.45,347.82)	-2.82(-3.40,-2.24)
Rwanda	3323.65(1762.46,5110.76)	514.59(265.35,797.38)	3364.56(1855.36,5164.91)	258.23(139.64,397.14)	-3.20(-3.60,-2.80)
Saint Kitts and Nevis	47.82(28.30,64.69)	477.23(277.34,648.14)	37.00(22.14,51.78)	244.64(140.62,344.69)	-2.13(-2.32,-1.93)
Saint Lucia	84.86(62.32,103.96)	417.23(304.05,514.09)	108.30(64.54,152.19)	171.53(101.62,241.64)	-3.50(-3.93,-3.07)

Saint Vincent and the Grenadines	48.06(25.79,67.94)	263.10(136.18,377.14)	59.56(36.24,82.33)	164.51(97.73,230.20)	-1.41(-1.63,-1.19)
Samoa	66.65(39.76,94.87)	336.20(192.50,488.33)	98.52(57.03,144.26)	286.80(164.51,420.42)	-0.56(-0.66,-0.46)
San Marino	15.34(9.56,21.27)	147.85(91.93,205.09)	12.69(6.68,20.15)	44.01(23.41,70.05)	-3.38(-3.75,-3.02)
Sao Tome and Principe	46.05(27.45,64.09)	290.26(169.75,406.59)	76.44(49.85,104.86)	310.86(200.61,427.04)	0.30(0.15,0.45)
Saudi Arabia	3857.50(2195.99,5666.70)	315.34(179.01,461.62)	6454.32(4116.59,9250.73)	199.64(129.10,280.34)	-1.72(-1.87,-1.58)
Senegal	2899.16(1930.63,3956.97)	388.94(257.39,532.21)	6008.63(4008.63,8389.17)	348.40(232.70,484.10)	-0.35(-0.40,-0.30)
Serbia	17358.58(12259.40,21787.98)	770.12(529.98,973.74)	18153.92(13071.59,23345.46)	377.81(271.60,486.75)	-2.93(-3.22,-2.65)
Seychelles	42.80(28.34,56.45)	279.70(184.85,369.12)	47.95(30.43,66.00)	171.02(107.03,235.97)	-1.17(-1.41,-0.93)
Sierra Leone	2079.72(1425.20,2769.58)	426.59(292.28,566.47)	3159.57(2099.58,4481.04)	373.53(249.42,525.70)	-0.25(-0.43,-0.07)
Singapore	972.16(646.78,1265.65)	190.81(123.30,251.25)	499.21(252.75,762.03)	22.13(11.19,33.79)	-6.99(-7.27,-6.72)
Slovakia	5553.73(3807.51,7164.01)	350.73(238.79,453.64)	3964.92(2516.25,5373.80)	150.21(94.99,203.83)	-2.84(-2.93,-2.75)
Slovenia	1804.91(1271.55,2240.55)	268.75(188.55,334.96)	1121.23(706.40,1493.51)	74.74(47.56,99.28)	-4.23(-4.37,-4.08)
Solomon Islands	103.32(44.30,172.09)	365.59(154.81,602.69)	269.45(130.90,423.75)	380.68(188.12,587.07)	0.12(0.02,0.22)
Somalia	1744.85(841.66,2896.31)	349.48(166.73,568.07)	3753.34(2001.28,5911.44)	305.15(161.85,477.37)	-0.37(-0.43,-0.31)
South Africa	9293.19(6052.18,12197.13)	190.94(124.12,251.72)	24471.59(17482.90,30664.44)	233.85(165.93,294.52)	0.67(0.11,1.23)
South Korea	27708.49(19180.36,35649.69)	473.82(325.77,612.56)	16608.69(8801.43,25035.05)	65.66(34.28,99.65)	-7.33(-7.70,-6.95)
South Sudan	1747.76(863.65,2797.31)	283.50(138.80,454.01)	2153.99(1185.20,3354.14)	261.52(146.08,401.92)	-0.44(-0.64,-0.23)
Spain	32566.50(24448.49,39720.55)	221.63(165.44,271.72)	16925.66(10465.53,23229.27)	47.45(29.82,64.47)	-4.86(-4.99,-4.74)
Sri Lanka	7534.37(4886.55,10272.64)	353.19(229.90,481.38)	15117.49(8864.76,22022.14)	237.99(139.77,345.86)	-0.58(-0.89,-0.27)
Sudan	8861.25(5453.47,12720.50)	418.08(255.94,601.81)	11657.33(7292.80,16534.55)	276.13(173.71,388.93)	-1.52(-1.58,-1.46)
Suriname	144.99(76.15,211.27)	230.36(121.76,333.90)	265.65(141.74,414.76)	160.45(84.55,251.29)	-1.11(-1.39,-0.83)
Swaziland	226.72(134.33,330.50)	376.99(219.34,553.86)	428.59(251.16,653.13)	363.85(208.89,550.48)	0.37(-0.12,0.87)

Sweden	6920.33(4819.46,8849.11)	146.63(102.04,187.71)	3897.32(2428.96,5312.25)	51.10(32.13,69.33)	-3.55(-3.79,-3.31)
Switzerland	3796.37(2354.70,5116.01)	118.24(73.28,159.49)	2151.85(1168.21,3117.86)	32.75(18.26,46.93)	-4.00(-4.11,-3.89)
Syria	3284.21(1899.14,4801.75)	283.56(165.10,412.48)	5862.30(3519.70,8607.06)	212.67(127.85,309.34)	-1.43(-1.68,-1.18)
Taiwan (Province of China)	10093.60(7183.92,12589.14)	282.65(200.50,353.83)	6898.07(3918.63,9782.15)	57.72(32.89,81.74)	-5.25(-5.56,-4.95)
Tajikistan	2177.79(1306.92,3065.48)	322.48(192.00,455.12)	3283.23(2031.57,4585.30)	285.67(178.50,395.50)	-0.64(-1.08,-0.19)
Tanzania	6204.09(3968.97,8575.77)	250.61(158.89,348.66)	12194.77(7034.62,18084.16)	216.72(122.18,322.34)	-0.45(-0.55,-0.34)
Thailand	16139.89(9313.38,23767.85)	200.69(114.70,296.72)	34335.96(19305.39,51878.34)	114.70(64.60,173.03)	-2.30(-2.56,-2.04)
The Bahamas	66.33(38.54,91.28)	176.50(102.08,243.14)	109.83(64.05,156.80)	111.19(64.45,158.68)	-1.53(-1.63,-1.42)
The Gambia	272.38(179.07,384.83)	356.70(235.72,500.59)	860.64(553.74,1212.74)	391.19(250.21,551.37)	0.28(0.17,0.38)
Timor-Leste	213.26(117.65,314.10)	375.03(208.13,549.25)	769.04(473.54,1087.32)	375.40(227.94,534.46)	0.24(0.06,0.41)
Togo	1038.33(668.01,1425.18)	391.67(251.65,536.44)	3014.11(1960.26,4266.23)	387.29(254.33,541.11)	-0.03(-0.20,0.14)
Tokelau	1.09(0.49,1.72)	310.39(136.49,495.17)	1.01(0.55,1.50)	245.50(134.09,366.16)	-0.91(-1.05,-0.77)
Tonga	19.61(9.58,30.80)	149.82(70.97,238.62)	33.44(19.08,49.43)	160.64(91.17,237.93)	0.40(0.23,0.57)
Trinidad and Tobago	551.71(364.54,721.08)	277.26(175.71,371.20)	744.24(464.86,1062.10)	145.08(90.11,207.43)	-2.45(-2.69,-2.20)
Tunisia	2383.78(1385.34,3520.84)	225.77(128.27,338.29)	4787.92(2537.85,7621.04)	150.06(79.36,239.59)	-1.55(-1.66,-1.45)
Turkey	22370.34(14229.19,30889.03)	289.84(184.21,399.78)	32672.02(20577.09,46252.07)	143.87(90.30,203.78)	-2.25(-2.49,-2.00)
Turkmenistan	1270.69(783.32,1692.01)	273.98(167.18,366.34)	3511.93(2270.24,4834.83)	364.61(237.63,496.96)	0.68(0.18,1.19)
Tuvalu	5.96(2.56,9.66)	389.32(159.17,638.59)	8.33(4.69,12.06)	335.86(187.70,489.47)	-0.53(-0.63,-0.42)
Uganda	5097.34(3245.84,7207.23)	350.79(222.30,496.63)	6795.22(4279.08,9910.60)	215.93(136.28,314.56)	-2.28(-2.60,-1.97)
Ukraine	77777.10(51189.46,99113.38)	430.09(277.76,553.41)	51309.02(31358.06,72424.41)	234.69(143.57,331.92)	-2.64(-2.93,-2.34)
United Arab Emirates	237.29(157.67,331.82)	315.43(211.68,432.97)	633.38(376.14,932.67)	181.42(111.01,259.26)	0.10(-0.52,0.72)
United Kingdom	52147.46(39973.65,61131.02)	197.17(150.59,231.89)	20583.38(14511.09,26069.07)	48.17(34.10,60.77)	-4.96(-5.22,-4.69)

United States	87286.78(62811.86,107119.21)	94.96(68.12,116.76)	99771.20(62736.56,135980.88)	56.59(35.81,76.82)	-2.24(-2.56,-1.92)
Uruguay	2548.81(1592.82,3383.39)	241.85(150.73,321.67)	1976.30(1204.75,2656.06)	109.70(67.43,146.97)	-2.82(-3.05,-2.58)
Uzbekistan	7231.55(4833.01,9445.83)	245.47(163.93,320.50)	12672.90(8148.37,17194.54)	217.38(135.35,297.68)	-0.95(-1.43,-0.48)
Vanuatu	64.81(43.36,89.93)	499.35(336.02,686.89)	152.53(99.74,206.96)	406.83(264.57,553.34)	-0.82(-0.88,-0.75)
Venezuela	3930.41(2891.98,4807.58)	170.89(125.22,209.59)	9432.03(5919.37,13551.82)	124.22(78.49,177.59)	-1.56(-1.84,-1.29)
Vietnam	45057.55(27216.59,64845.70)	460.84(278.95,662.18)	104751.18(70251.57,140057.55)	452.83(303.99,602.39)	0.32(0.13,0.51)
Virgin Islands, U.S.	28.13(15.22,41.75)	154.39(80.96,231.00)	33.11(18.96,49.65)	67.10(37.48,101.65)	-2.46(-2.62,-2.30)
Yemen	4224.80(2184.71,6616.76)	411.76(210.31,643.49)	9325.28(5088.02,14435.01)	317.87(172.19,492.06)	-1.09(-1.23,-0.95)
Zambia	1890.98(1074.18,2823.88)	307.16(174.93,457.17)	4570.27(2484.14,6947.45)	312.26(165.56,477.02)	-0.17(-0.31,-0.03)
Zimbabwe	2161.70(1452.98,2944.23)	255.01(170.58,347.88)	5517.19(3624.31,7784.40)	382.28(250.84,531.34)	2.07(1.42,2.73)

Table S2. Age-standardized DALYs Rate of Stroke Caused by High Systolic Blood Pressure Among People Aged 45 and Above by Country.

**Location**	**1990**		**2021**		**1990-2021**
	**DALYs cases**	**DALYs rate**	**DALYs cases**	**DALYs rate**	**EAPC**
Afghanistan	185759.64(96084.28,283342.52)	9924.83(5142.12,15089.33)	174613.73(88735.90,275159.65)	7069.53(3673.28,10943.65)	-1.37(-1.57,-1.17)
Albania	38791.55(27577.77,49502.24)	7864.54(5578.32,10020.42)	61053.59(40673.69,82216.52)	5142.50(3402.74,6943.86)	-1.21(-1.43,-0.98)
Algeria	157494.39(99133.70,223656.90)	5979.95(3812.51,8440.92)	280351.98(164069.63,415397.50)	3492.18(2067.88,5136.45)	-1.82(-1.87,-1.76)
American Samoa	341.72(199.83,480.20)	5774.33(3264.41,8266.90)	643.95(399.89,899.85)	4947.27(3039.13,6933.69)	-0.75(-0.89,-0.61)

Andorra	237.25(143.74,355.57)	1635.99(977.34,2457.38)	358.91(205.00,542.02)	797.50(460.62,1198.60)	-2.16(-2.37,-1.95)
Angola	81487.34(46997.54,120458.37)	8402.05(4869.19,12289.45)	171352.54(101355.88,250439.02)	6085.20(3632.10,8849.89)	-1.34(-1.44,-1.24)
Antigua and Barbuda	668.05(392.11,923.28)	4510.08(2624.09,6257.93)	760.60(462.24,1037.68)	2682.76(1616.25,3672.56)	-2.01(-2.24,-1.77)
Argentina	318837.37(176300.42,452274.81)	3713.52(2051.40,5260.97)	269798.37(179417.22,353250.02)	1746.19(1160.40,2289.11)	-2.11(-2.21,-2.01)
Armenia	34193.92(20959.80,44566.38)	4909.84(2992.85,6425.27)	33654.46(23246.66,43384.00)	2831.11(1942.24,3658.94)	-2.64(-3.00,-2.27)
Australia	127937.81(95392.14,157007.71)	2435.92(1804.72,3001.49)	103818.50(68024.40,139959.49)	776.85(510.09,1043.14)	-3.96(-4.16,-3.77)
Austria	112234.45(74481.68,144223.92)	3285.29(2189.53,4223.97)	53697.96(33684.10,72210.31)	973.00(616.33,1303.36)	-4.17(-4.48,-3.85)
Azerbaijan	71177.02(42613.03,98536.76)	5417.18(3212.75,7519.11)	109963.27(69435.62,152216.47)	4414.60(2813.57,6055.66)	-0.53(-0.78,-0.28)
Bahrain	2081.98(1278.35,2845.03)	5792.80(3555.69,7934.59)	4654.34(2814.29,6563.58)	2715.20(1668.80,3815.83)	-3.16(-3.59,-2.73)
Bangladesh	884926.24(510648.39,1291899.12)	7336.62(4263.94,10671.35)	2039578.70(1285896.64,2878090.33)	5670.07(3575.72,7996.33)	-0.68(-0.79,-0.58)
Barbados	3148.04(2188.21,4052.59)	3857.39(2662.11,4997.82)	3922.53(2625.85,5316.87)	2729.88(1819.78,3709.28)	-1.32(-1.49,-1.14)
Belarus	224271.66(150240.48,283810.71)	6316.31(4199.99,8015.61)	205188.15(142520.10,268477.26)	4611.85(3201.50,6041.88)	-1.76(-2.23,-1.30)
Belgium	116438.75(75719.66,151802.30)	2668.27(1732.89,3487.36)	75695.72(52196.42,96986.24)	1071.44(745.22,1365.92)	-2.88(-2.93,-2.82)
Belize	683.92(409.53,926.40)	2752.71(1650.05,3726.19)	1616.66(986.04,2224.21)	2075.21(1276.27,2843.13)	-1.39(-1.91,-0.87)
Benin	40618.09(23625.92,57378.47)	7919.51(4604.96,11181.18)	83709.19(55011.79,115837.73)	6549.65(4268.23,9052.74)	-0.56(-0.67,-0.44)
Bermuda	439.78(260.66,607.64)	2678.36(1572.33,3715.13)	443.48(275.27,612.58)	1132.30(702.54,1565.88)	-2.91(-3.06,-2.76)
Bhutan	2662.00(1418.88,4188.88)	4315.41(2265.45,6822.90)	4482.30(2690.78,6566.34)	2822.72(1682.95,4138.87)	-1.45(-1.52,-1.38)
Bolivia	28881.90(11288.36,51948.70)	3489.98(1380.30,6218.02)	50439.83(22765.79,87720.72)	2151.35(988.51,3696.88)	-1.47(-1.65,-1.30)
Bosnia and Herzegovina	74071.82(51549.86,93943.11)	7318.67(5042.25,9304.38)	79932.42(52164.44,106077.14)	4538.57(2958.76,6037.60)	-1.83(-1.98,-1.68)
Botswana	11329.93(6875.25,16282.26)	8147.73(4885.39,11760.83)	17155.61(10831.38,24426.47)	4821.81(3001.19,6878.14)	-1.66(-1.91,-1.42)
Brazil	1270399.22(926761.83,1557319.26)	5477.57(3994.88,6721.61)	1467797.52(1077473.83,1792489.49)	2145.45(1573.51,2622.37)	-3.07(-3.12,-3.01)
Brunei	1595.19(996.78,2200.00)	6438.48(4024.17,8852.02)	2386.89(1510.16,3334.47)	2777.20(1690.20,3930.23)	-2.71(-2.96,-2.45)
Bulgaria	384243.25(278372.85,471650.73)	12379.53(8858.85,15253.09)	300438.60(199978.89,392647.03)	7482.23(4939.43,9816.55)	-1.97(-2.15,-1.79)
Burkina Faso	54595.32(31441.90,81528.40)	4869.80(2808.97,7260.01)	104248.31(62366.40,151148.76)	4478.60(2688.84,6457.60)	-0.03(-0.11,0.05)

Burundi	62550.90(32166.35,96856.69)	10212.85(5246.57,15811.11)	74497.12(43613.07,109062.67)	6132.49(3623.95,8968.36)	-2.14(-2.37,-1.91)
Cambodia	100607.70(51703.24,152805.64)	8931.65(4658.81,13408.45)	182488.91(94710.79,280663.33)	6137.74(3245.33,9290.26)	-1.42(-1.55,-1.28)
Cameroon	63219.44(39058.51,92242.11)	5583.62(3474.81,8096.25)	224332.74(142134.36,327144.97)	7097.08(4558.48,10241.80)	0.86(0.28,1.45)
Canada	162218.29(115866.94,204273.63)	1835.53(1305.51,2317.42)	163513.53(92568.95,232365.64)	772.51(434.82,1102.06)	-3.10(-3.30,-2.90)
Cape Verde	2686.63(1654.13,3766.34)	4296.85(2644.97,6031.57)	5617.23(3624.20,7770.55)	4783.55(3086.51,6603.58)	0.09(-0.20,0.37)
Central African Republic	29929.11(16309.65,46776.64)	10266.01(5694.50,15828.85)	46587.97(25491.15,74337.47)	8483.98(4684.88,13231.02)	-0.71(-0.79,-0.63)
Chad	47217.37(26296.09,69845.42)	6367.82(3527.23,9425.15)	101080.52(59888.39,150422.08)	6845.16(4072.56,10129.82)	0.18(-0.02,0.39)
Chile	113141.24(82116.47,139273.80)	4321.87(3136.69,5322.25)	117355.13(82493.53,149541.10)	1641.70(1152.47,2094.67)	-2.89(-2.99,-2.78)
China	16074695.39(9072388.27,23346316.39)	7895.45(4445.31,11431.51)	29223546.40(18096448.93,40512714.06)	5147.75(3185.96,7145.27)	-1.46(-1.71,-1.21)
Colombia	136194.32(86515.93,180017.69)	3046.20(1936.61,4015.68)	201104.11(137190.03,267139.46)	1329.25(910.45,1762.40)	-3.13(-3.30,-2.96)
Comoros	3834.31(2143.21,5792.14)	7447.88(4114.52,11228.62)	6248.31(3832.84,9027.11)	4981.98(3052.39,7174.65)	-1.58(-1.78,-1.39)
Congo	27015.71(16427.37,38882.44)	9928.74(6027.98,14208.51)	43681.53(26702.96,63767.86)	6626.93(4077.07,9509.45)	-1.63(-1.77,-1.48)
Cook Islands	140.32(68.12,219.54)	4276.40(2068.75,6693.88)	200.60(124.20,284.97)	2795.22(1722.50,3982.62)	-1.45(-1.61,-1.30)
Costa Rica	9120.57(6253.02,11650.67)	2004.87(1377.39,2559.52)	17683.88(12077.62,23029.28)	1173.51(802.96,1525.60)	-2.22(-2.54,-1.91)
Cote d'Ivoire	74234.44(45079.39,106530.67)	7305.33(4406.42,10443.99)	187098.88(117458.52,267619.59)	6629.54(4192.62,9434.24)	-0.38(-0.58,-0.18)
Croatia	129752.78(92268.76,159955.01)	8410.65(5971.34,10373.94)	69401.17(46992.63,90095.09)	2629.51(1777.67,3420.02)	-3.98(-4.13,-3.83)
Cuba	75461.63(47275.39,102020.77)	2752.57(1720.89,3725.89)	103224.41(61009.71,145018.58)	1862.44(1102.58,2613.88)	-1.50(-1.64,-1.36)
Cyprus	8749.83(5588.41,12246.69)	5293.24(3273.63,7454.86)	6959.45(4165.41,9854.00)	1366.94(784.30,1955.61)	-4.67(-4.92,-4.42)
Czech Republic	294154.38(199109.62,371558.18)	7778.62(5270.34,9835.15)	105137.88(67361.57,138326.53)	1699.96(1093.16,2235.22)	-5.06(-5.29,-4.84)
Democratic Republic of the Congo	280323.84(174626.50,408661.73)	7198.65(4524.22,10441.76)	509159.43(301855.76,774798.41)	5766.91(3447.98,8771.64)	-0.84(-0.96,-0.72)
Denmark	74194.41(56689.46,89692.17)	3183.69(2419.94,3858.34)	41926.42(27219.48,55134.41)	1190.13(773.41,1564.22)	-3.52(-3.73,-3.31)
Djibouti	2090.36(1078.37,3312.72)	6236.48(3243.62,9773.65)	8514.33(4894.99,12997.99)	5458.01(3210.77,8210.76)	-0.54(-0.62,-0.45)
Dominica	729.98(431.20,1016.34)	4520.35(2646.79,6330.06)	768.23(441.35,1112.10)	3404.02(1935.60,4939.97)	-0.91(-1.05,-0.76)

Dominican Republic	31724.94(17093.37,46694.66)	3337.97(1806.26,4892.35)	81754.85(46686.10,123372.65)	3013.37(1724.23,4541.63)	0.15(-0.02,0.31)
Ecuador	26498.45(11569.61,42499.66)	1928.34(841.55,3084.68)	56556.07(29690.43,85996.15)	1302.24(685.60,1975.73)	-1.03(-1.30,-0.77)
Egypt	496843.75(273454.68,725621.01)	8185.65(4517.12,11922.46)	973173.34(627882.49,1362354.42)	6625.14(4195.51,9277.16)	-0.25(-0.43,-0.06)
El Salvador	19236.87(10899.73,27586.20)	2437.03(1387.55,3486.00)	23082.08(13405.07,34222.99)	1348.41(785.22,1999.90)	-2.10(-2.48,-1.71)
Equatorial Guinea	4814.91(2770.87,7215.50)	9567.99(5557.91,14216.05)	5685.36(3192.63,9104.57)	4773.64(2722.37,7524.29)	-2.67(-3.02,-2.31)
Eritrea	26796.52(13717.17,42340.94)	9057.91(4537.03,14121.42)	42804.19(23895.92,65281.70)	6411.83(3634.75,9604.94)	-1.07(-1.19,-0.95)
Estonia	42333.72(29349.95,52658.33)	7518.38(5183.15,9383.96)	12891.50(8799.47,16698.33)	1674.44(1152.99,2161.55)	-6.19(-6.72,-5.66)
Ethiopia	204691.88(102312.12,337592.58)	3882.14(1923.71,6377.09)	319805.95(194322.41,451019.10)	2888.32(1719.54,4114.32)	-1.24(-1.34,-1.15)
Federated States of Micronesia	1135.70(653.70,1675.86)	8995.88(5070.18,13421.26)	1405.61(727.79,2200.64)	6940.13(3537.24,10856.01)	-0.86(-0.94,-0.79)
Fiji	5675.51(3175.96,8353.05)	6065.76(3315.43,9006.38)	11717.92(7544.27,16501.17)	5848.90(3688.56,8243.77)	-0.29(-0.45,-0.13)
Finland	76198.06(59322.34,90819.07)	3837.18(2971.48,4588.93)	49266.11(32209.57,65204.90)	1304.27(866.78,1711.02)	-3.47(-3.60,-3.34)
France	543849.88(394267.88,683442.08)	2283.37(1654.94,2871.50)	382900.24(233928.31,518823.96)	864.51(535.24,1163.66)	-3.15(-3.25,-3.04)
Gabon	10512.71(6329.49,15169.93)	7048.18(4227.95,10167.98)	13291.19(7933.61,19644.49)	5155.16(3094.09,7555.58)	-1.14(-1.28,-1.00)
Georgia	155719.37(112638.06,191242.11)	9142.54(6528.28,11297.55)	116656.87(79625.64,149366.00)	6892.40(4710.66,8809.24)	-1.21(-1.52,-0.89)
Germany	1362842.62(1041449.90,1632874.25)	3712.47(2827.46,4460.61)	711073.22(482956.67,921301.06)	1206.81(815.71,1566.48)	-3.78(-4.08,-3.48)
Ghana	151526.93(100816.52,208270.17)	9435.87(6313.51,12887.73)	361999.39(247070.06,495457.23)	8585.11(5892.94,11659.53)	0.01(-0.19,0.22)
Greece	220754.77(165298.64,267158.79)	5309.44(3952.23,6457.65)	124638.60(77960.78,168509.64)	1611.75(1014.75,2170.72)	-4.53(-4.86,-4.20)
Greenland	506.03(280.47,720.21)	6673.56(3810.14,9381.72)	396.40(208.38,604.89)	2336.09(1245.36,3540.90)	-3.58(-3.72,-3.43)
Grenada	1226.12(696.67,1707.82)	6185.00(3463.08,8664.05)	1001.09(590.12,1371.00)	3319.39(1909.79,4575.58)	-1.96(-2.11,-1.81)
Guam	562.81(259.98,855.79)	3050.99(1371.87,4694.42)	1268.66(786.74,1752.83)	2164.62(1343.55,2989.78)	-0.95(-1.22,-0.69)
Guatemala	17176.22(9055.71,24863.92)	2017.96(1072.82,2918.57)	44968.54(28634.70,61168.50)	1553.68(973.22,2129.51)	-1.33(-1.64,-1.03)
Guinea	55794.73(31752.48,82545.67)	6397.16(3633.82,9462.82)	98392.48(59746.78,143941.91)	6759.02(4095.01,9854.84)	0.50(0.33,0.67)
Guinea-Bissau	11967.20(6782.47,18101.96)	11475.84(6551.13,17164.28)	18685.90(11314.99,26971.96)	10269.54(6276.13,14598.54)	-0.19(-0.28,-0.11)
Guyana	11222.26(6358.42,15561.76)	11228.41(6400.35,15512.88)	9422.12(5374.72,13904.69)	5493.39(3128.65,8075.31)	-1.84(-2.06,-1.63)

Haiti	89647.85(51300.41,131166.61)	10624.85(6034.43,15576.77)	151488.07(91828.97,221438.14)	8069.39(4802.57,11826.57)	-0.68(-0.75,-0.60)
Honduras	19374.53(11829.67,27104.91)	3660.33(2235.83,5116.81)	75037.21(44989.01,109672.85)	4602.09(2763.47,6690.21)	0.95(0.74,1.16)
Hungary	360158.61(283470.69,420845.92)	9069.65(7120.30,10617.71)	150764.75(106493.67,190920.41)	2721.29(1918.31,3454.99)	-4.26(-4.45,-4.07)
Iceland	1712.03(1139.87,2242.20)	2102.11(1403.13,2750.89)	1380.16(877.48,1863.05)	792.97(505.60,1067.36)	-3.23(-3.37,-3.09)
India	4885185.33(3488048.36,6196064.96)	4008.29(2857.50,5088.97)	9964764.98(7263393.83,12465281.91)	3112.66(2267.43,3896.14)	-0.91(-0.99,-0.83)
Indonesia	2599003.57(1927412.49,3211654.63)	10306.31(7607.24,12806.16)	6515996.84(4828582.64,8193038.00)	10670.06(7935.78,13292.43)	0.22(0.06,0.38)
Iran	269145.41(194197.62,331978.79)	4396.40(3156.39,5451.79)	445640.02(322533.77,550511.64)	2282.07(1647.97,2828.41)	-2.24(-2.36,-2.11)
Iraq	190682.95(123024.87,262496.64)	9186.32(5941.12,12620.39)	434787.28(288528.89,594806.30)	7416.88(4980.22,10020.26)	-1.25(-1.42,-1.08)
Ireland	36921.51(26034.55,46162.06)	3280.84(2278.45,4138.68)	18063.98(11821.11,23884.35)	791.47(517.31,1047.42)	-4.71(-4.92,-4.50)
Israel	33550.16(25221.74,41065.93)	2543.28(1893.46,3134.69)	31459.66(21392.33,40830.67)	885.60(602.66,1148.70)	-3.83(-4.02,-3.64)
Italy	839958.83(631642.15,1006011.98)	3375.50(2520.20,4064.29)	423239.94(255518.78,584339.32)	893.15(548.71,1221.54)	-4.60(-4.77,-4.44)
Jamaica	19975.60(13018.22,26495.40)	4048.52(2623.86,5389.36)	28346.78(16896.65,40417.13)	3315.28(1993.90,4707.62)	-0.32(-0.72,0.09)
Japan	1645597.33(1256551.71,1962742.66)	3644.68(2768.50,4361.71)	1372907.59(906535.66,1837054.13)	1309.79(885.93,1718.41)	-3.57(-3.76,-3.38)
Jordan	18057.51(11216.86,25445.82)	5759.92(3633.57,8072.31)	44997.64(28526.42,62594.12)	2609.37(1665.32,3619.21)	-3.12(-3.49,-2.74)
Kazakhstan	281560.58(195972.61,356798.13)	8611.90(5985.54,10896.62)	325078.67(234870.84,408588.51)	7349.92(5324.03,9200.30)	-1.08(-1.52,-0.63)
Kenya	83289.16(49926.04,118620.44)	4068.92(2446.82,5766.90)	238244.47(159797.33,322208.26)	4271.85(2833.73,5790.33)	0.43(0.24,0.61)
Kiribati	710.93(349.27,1114.98)	7203.48(3489.13,11362.47)	1330.32(706.69,2073.93)	6997.22(3747.25,10853.16)	-0.18(-0.27,-0.09)
Kuwait	3016.89(1958.71,3995.25)	2194.69(1465.36,2869.62)	9458.26(6359.25,12871.79)	1357.78(922.40,1827.93)	-1.39(-2.36,-0.40)
Kyrgyzstan	64097.18(38102.86,87664.53)	8149.97(4812.75,11173.35)	53330.33(30817.24,75219.82)	4208.48(2503.89,5845.77)	-2.76(-3.30,-2.21)
Laos	71335.23(40702.45,106457.21)	12885.11(7322.56,19178.90)	91625.34(54913.56,133109.64)	7682.75(4593.05,11113.03)	-1.82(-1.90,-1.75)
Latvia	83735.71(56486.50,105779.02)	8458.64(5690.66,10708.85)	52059.92(35218.62,67254.18)	4409.19(3007.02,5681.78)	-2.77(-3.10,-2.44)
Lebanon	28516.35(16338.48,42554.72)	5107.95(2927.68,7625.74)	28311.12(18316.71,39069.07)	1675.93(1090.50,2309.33)	-3.75(-4.07,-3.43)
Lesotho	12917.29(7192.59,19379.12)	5952.38(3283.52,8962.45)	25327.68(14221.55,39148.49)	9205.69(5066.36,14155.07)	2.28(1.75,2.80)
Liberia	21431.99(13020.52,30484.07)	7216.42(4383.10,10258.80)	35841.46(22283.19,51769.92)	6775.13(4254.85,9772.42)	-0.32(-0.45,-0.18)
Libya	16108.29(10177.55,23333.45)	3378.48(2140.92,4886.57)	45090.04(27366.01,67003.30)	3350.90(2035.18,4972.85)	0.24(0.07,0.41)

Lithuania	58987.94(42061.27,73306.88)	4740.95(3368.66,5899.43)	51022.73(34600.43,65368.53)	3072.85(2108.43,3920.65)	-1.42(-1.73,-1.10)
Luxembourg	6732.33(4509.66,8525.56)	4468.14(2965.60,5691.07)	2984.82(1928.16,3959.94)	957.63(625.67,1264.19)	-4.98(-5.09,-4.86)
Macedonia	57712.51(40314.04,72828.37)	12314.35(8630.45,15507.42)	74846.26(48938.64,100361.78)	9466.63(6083.88,12765.64)	-1.14(-1.49,-0.79)
Madagascar	122310.72(64490.55,182435.98)	9180.01(4839.30,13692.79)	253514.42(145028.26,380929.51)	8870.36(5122.60,13155.17)	-0.11(-0.18,-0.05)
Malawi	72328.25(44792.95,101243.64)	7209.68(4360.03,10196.33)	134006.42(89000.87,183801.43)	7225.84(4731.68,9909.72)	-0.31(-0.61,-0.01)
Malaysia	166997.06(119220.22,211783.21)	6956.91(4992.07,8792.75)	362939.76(264483.50,464547.43)	4860.91(3531.95,6233.38)	-0.91(-1.11,-0.70)
Maldives	2526.19(1638.30,3394.95)	10353.47(6562.95,14032.05)	2710.73(1750.76,3674.34)	3277.33(2105.13,4443.39)	-4.17(-4.34,-4.00)
Mali	58738.94(31832.01,87521.53)	5891.89(3249.36,8683.26)	104584.01(60339.73,155590.05)	4848.14(2859.81,7109.00)	-0.48(-0.61,-0.34)
Malta	3919.74(2713.18,4952.67)	3431.53(2357.44,4353.44)	2482.70(1518.67,3386.38)	866.71(533.23,1178.67)	-4.54(-4.71,-4.36)
Marshall Islands	344.81(171.54,543.99)	8314.60(4036.87,13235.55)	688.57(342.89,1069.03)	7616.00(3916.80,11652.58)	-0.27(-0.38,-0.16)
Mauritania	22483.49(13871.00,31963.46)	8619.15(5260.58,12279.64)	32058.53(19339.50,47828.77)	5827.69(3531.94,8658.64)	-1.38(-1.55,-1.21)
Mauritius	15474.16(11366.92,19263.03)	8082.24(5924.72,10071.29)	15780.39(10549.97,20331.15)	3155.26(2102.66,4073.51)	-4.26(-4.86,-3.65)
Mexico	220082.25(139400.12,293720.43)	2092.61(1332.94,2798.59)	434137.72(289199.44,568395.98)	1282.81(855.02,1680.48)	-1.66(-1.75,-1.58)
Moldova	89295.65(60207.32,112897.00)	7779.43(5162.97,9898.03)	75295.83(54257.99,93774.92)	4545.72(3266.07,5670.72)	-2.13(-2.44,-1.81)
Monaco	808.10(504.73,1127.04)	3766.69(2344.80,5296.08)	447.55(261.01,647.65)	1466.63(859.70,2129.24)	-3.17(-3.36,-2.99)
Mongolia	28036.00(18851.65,37417.44)	10117.13(6811.18,13501.03)	41920.42(28575.42,55852.35)	6663.95(4489.50,8913.15)	-1.61(-2.15,-1.07)
Montenegro	13041.34(8894.47,16802.59)	7925.25(5402.41,10212.80)	20003.88(12961.73,26836.60)	7847.93(5057.51,10562.45)	-0.16(-0.37,0.05)
Morocco	251586.32(164977.50,350530.78)	6850.60(4492.88,9525.13)	478767.45(305888.62,675981.67)	5405.84(3464.96,7609.55)	-0.75(-0.77,-0.73)
Mozambique	148894.49(95075.96,205305.12)	9202.35(5729.70,12851.77)	332839.76(211580.67,466439.86)	11192.62(7037.02,15689.67)	1.10(0.87,1.34)
Myanmar	800795.16(495715.78,1122780.34)	13062.35(8148.99,18240.00)	1028244.59(670347.48,1441368.03)	7924.31(5161.08,11104.89)	-1.88(-2.05,-1.70)
Namibia	13023.68(7729.68,18396.71)	8081.15(4757.18,11446.73)	21592.04(12797.30,31504.30)	6384.79(3728.38,9305.33)	-1.04(-1.34,-0.73)
Nauru	153.80(100.41,217.34)	12633.92(8313.61,17740.30)	181.68(115.48,262.71)	11554.55(7212.33,16649.01)	-0.44(-0.87,-0.00)
Nepal	114137.39(61762.79,173867.91)	4628.49(2477.95,7068.43)	169544.86(95313.49,259037.75)	2735.79(1524.65,4193.21)	-1.64(-1.72,-1.56)
Netherlands	138941.75(101057.00,172466.91)	2473.20(1792.29,3079.64)	113545.24(70625.10,151905.47)	1087.29(675.47,1455.52)	-3.33(-3.68,-2.97)
New Zealand	27965.96(21108.71,33842.84)	2607.65(1958.20,3167.34)	23148.70(15503.59,30554.62)	945.96(633.54,1247.43)	-3.41(-3.57,-3.26)

Nicaragua	9992.48(6063.42,13701.78)	2535.31(1536.68,3480.71)	18497.67(11408.96,26464.22)	1446.75(889.12,2071.60)	-1.76(-1.92,-1.60)
Niger	43644.52(23966.06,66159.70)	6314.20(3481.81,9500.94)	121512.21(69533.33,182559.50)	5944.18(3443.64,8859.68)	-0.06(-0.13,0.01)
Nigeria	692501.45(430082.99,961563.21)	6116.41(3812.69,8473.27)	1036309.76(727910.96,1386361.10)	4627.55(3272.10,6135.48)	-0.88(-1.02,-0.74)
Niue	43.81(24.99,64.13)	7081.88(4033.68,10392.03)	35.09(23.23,48.15)	5893.68(3900.24,8081.09)	-0.87(-0.98,-0.77)
North Korea	382979.74(215988.61,559970.21)	9035.50(5016.25,13295.15)	703408.31(399026.64,1030901.59)	7814.05(4413.71,11458.09)	-0.52(-0.73,-0.32)
Northern Mariana Islands	219.81(107.86,348.83)	5180.60(2591.98,8063.64)	585.78(356.08,820.10)	4253.78(2590.67,5949.88)	-0.83(-1.02,-0.65)
Norway	63027.13(48174.02,75179.22)	3120.75(2384.55,3725.41)	28551.27(19194.80,37246.93)	943.71(637.67,1227.47)	-4.13(-4.25,-4.02)
Oman	7169.61(3661.67,11267.97)	4216.85(2159.25,6631.30)	13741.78(8829.38,19357.07)	3018.82(1939.72,4250.94)	-0.40(-0.67,-0.14)
Pakistan	673712.89(445202.73,906691.18)	4563.52(3011.20,6146.13)	1393037.58(922976.89,1905308.06)	4425.20(2927.58,6052.06)	-0.39(-0.60,-0.19)
Palau	161.01(79.01,252.34)	6292.95(3057.23,9903.65)	360.93(214.97,530.05)	5879.15(3490.45,8577.71)	-0.20(-0.31,-0.08)
Palestine	13482.74(8272.64,19231.72)	6343.61(3853.58,9064.02)	19989.24(12054.13,28339.03)	3506.84(2107.56,4996.51)	-2.01(-2.30,-1.71)
Panama	11030.01(7062.37,14561.07)	2833.15(1816.31,3736.50)	21819.32(13438.90,30009.76)	1787.67(1103.96,2453.78)	-1.73(-1.91,-1.54)
Papua New Guinea	26754.82(13979.44,42395.04)	5963.71(3167.44,9362.66)	71500.42(33678.73,117543.81)	5355.49(2432.37,8877.78)	-0.31(-0.45,-0.18)
Paraguay	25677.20(17473.57,34131.53)	4412.05(2982.64,5879.37)	43615.44(25638.86,64203.52)	2831.32(1671.42,4153.83)	-1.40(-1.52,-1.29)
Peru	49022.84(22547.75,80918.11)	1563.47(723.29,2569.64)	107823.35(65833.95,160226.84)	1185.48(727.69,1757.18)	-1.13(-1.59,-0.67)
Philippines	361904.51(237446.91,481315.95)	4884.92(3221.86,6491.56)	1021127.46(650412.08,1383148.42)	4608.77(2943.86,6225.07)	-0.09(-0.26,0.08)
Poland	769818.30(542430.56,951756.46)	6625.20(4623.81,8237.59)	429729.92(283142.87,562876.67)	2134.41(1412.90,2790.23)	-4.03(-4.23,-3.83)
Portugal	283602.06(190815.52,362024.98)	7628.54(5051.37,9815.33)	106667.44(67822.14,142514.68)	1417.61(914.62,1880.00)	-5.88(-6.09,-5.67)
Puerto Rico	16379.12(9851.29,22218.25)	1700.72(1013.72,2314.86)	16074.15(9626.33,22481.34)	792.63(477.29,1104.84)	-2.92(-3.15,-2.70)
Qatar	1196.66(745.45,1707.06)	5851.02(3673.21,8218.57)	3738.69(2305.05,5382.90)	2041.30(1283.89,2901.41)	-3.82(-4.53,-3.11)
Romania	737949.71(559049.82,877297.98)	10252.96(7682.76,12277.67)	588997.13(434875.98,734254.95)	5457.76(4021.16,6824.48)	-2.78(-3.13,-2.43)
Russian Federation	4455889.76(3419882.96,5242903.98)	9436.09(7213.30,11143.70)	3505952.31(2622395.98,4211964.15)	5324.97(3978.98,6404.46)	-2.76(-3.35,-2.16)
Rwanda	85252.30(45389.44,131242.35)	11440.98(6018.14,17622.30)	79389.50(44786.99,120665.96)	5136.99(2862.38,7801.32)	-3.63(-4.06,-3.20)
Saint Kitts and Nevis	909.77(546.31,1222.83)	9000.44(5370.55,12102.39)	823.14(515.02,1138.53)	4594.14(2783.65,6385.22)	-2.26(-2.46,-2.05)

Saint Lucia	1632.44(1200.70,2001.21)	7335.93(5367.25,9024.82)	2005.16(1225.18,2781.91)	3066.92(1865.54,4262.58)	-3.25(-3.62,-2.87)
Saint Vincent and the Grenadines	923.89(504.59,1299.35)	4846.02(2596.66,6873.23)	1202.60(757.44,1630.67)	3117.75(1936.37,4259.96)	-1.38(-1.55,-1.21)
Samoa	1603.37(998.96,2240.68)	7208.72(4386.11,10190.60)	2372.48(1396.09,3448.72)	6163.52(3605.25,8955.15)	-0.55(-0.64,-0.47)
San Marino	247.99(158.58,338.12)	2445.96(1557.49,3338.49)	202.47(115.28,307.22)	844.85(485.71,1279.82)	-3.03(-3.32,-2.75)
Sao Tome and Principe	1050.82(648.58,1439.42)	6127.43(3735.87,8425.17)	1866.72(1246.46,2530.45)	6490.89(4301.33,8784.70)	0.15(-0.04,0.35)
Saudi Arabia	86896.69(50139.84,127984.61)	6102.64(3521.24,8934.78)	173614.93(110148.31,250180.27)	3777.95(2463.14,5313.01)	-1.77(-1.93,-1.61)
Senegal	69217.78(46225.51,94034.63)	8271.11(5521.74,11230.25)	134716.60(90664.00,187956.75)	6883.28(4652.56,9545.90)	-0.59(-0.66,-0.53)
Serbia	332206.49(241237.59,413396.24)	12836.18(9113.57,16056.82)	298556.31(216928.56,382690.44)	6299.45(4566.24,8098.31)	-2.92(-3.20,-2.64)
Seychelles	959.58(662.74,1239.57)	6292.45(4349.66,8124.26)	1121.48(734.81,1523.58)	3583.37(2325.08,4870.99)	-1.46(-1.66,-1.26)
Sierra Leone	47203.34(32409.02,63005.88)	8893.79(6112.02,11838.19)	74475.98(49498.78,105509.01)	7723.22(5170.77,10871.43)	-0.26(-0.45,-0.06)
Singapore	24291.12(16844.28,30914.89)	4286.03(2921.53,5497.44)	13936.08(7396.50,20812.29)	599.22(316.94,895.31)	-6.62(-6.80,-6.44)
Slovakia	112093.65(77355.00,143617.30)	6895.69(4739.02,8860.49)	78921.78(51640.04,105138.06)	2979.34(1942.23,3977.45)	-2.81(-2.90,-2.72)
Slovenia	33495.47(23609.44,41681.45)	4944.86(3478.78,6166.57)	17664.78(11544.43,23264.40)	1294.12(852.40,1700.45)	-4.52(-4.64,-4.40)
Solomon Islands	2767.49(1189.01,4651.50)	7934.12(3416.46,13158.55)	6892.56(3327.48,10966.52)	7904.35(3874.50,12344.99)	-0.00(-0.13,0.12)
Somalia	47706.12(23107.14,80030.77)	7825.64(3821.18,12827.13)	99695.74(52499.43,158550.05)	6616.48(3519.00,10373.06)	-0.51(-0.57,-0.45)
South Africa	220482.20(147276.07,284602.00)	4129.64(2756.23,5343.91)	545663.77(390843.79,682666.76)	4630.15(3312.34,5805.17)	0.38(-0.14,0.91)
South Korea	636452.61(440165.02,820939.75)	8982.89(6228.38,11567.92)	334570.59(184295.23,491279.87)	1293.74(706.12,1908.05)	-7.14(-7.50,-6.78)
South Sudan	41591.88(20545.51,66594.55)	6162.94(3039.85,9853.18)	54430.73(29996.41,85120.40)	5560.88(3128.13,8564.82)	-0.53(-0.76,-0.29)
Spain	525191.23(394847.34,640715.38)	3478.26(2601.08,4263.10)	279165.01(177988.24,375144.12)	921.88(593.72,1229.68)	-4.23(-4.37,-4.10)
Sri Lanka	153010.15(97492.69,209315.92)	6118.96(3939.32,8340.37)	291774.79(176361.41,422376.10)	4166.22(2515.72,6021.82)	-0.72(-1.00,-0.44)
Sudan	203998.99(125695.15,292352.01)	8512.63(5244.64,12198.68)	261504.94(164672.80,370526.94)	5367.56(3404.64,7548.36)	-1.68(-1.74,-1.62)
Suriname	3161.68(1654.84,4636.08)	4698.83(2479.79,6843.18)	5901.30(3296.67,9051.92)	3371.30(1865.54,5184.01)	-1.10(-1.39,-0.81)
Swaziland	5346.77(3191.29,7750.66)	7626.63(4533.35,11049.29)	10579.61(6325.66,16124.24)	7584.10(4490.38,11445.28)	0.44(-0.10,0.99)
Sweden	115760.55(83361.19,145876.20)	2578.24(1862.57,3244.91)	63463.23(40667.48,85274.01)	926.03(591.80,1246.31)	-3.52(-3.70,-3.34)

Switzerland	60053.69(38577.56,79153.91)	1952.63(1262.00,2569.36)	33931.01(19717.02,48222.93)	587.59(347.36,831.12)	-3.83(-3.93,-3.72)
Syria	73154.33(42088.17,107231.18)	5543.30(3226.34,8078.93)	131105.99(78312.82,193807.28)	3914.16(2353.68,5729.11)	-1.65(-1.92,-1.38)
Taiwan (Province of China)	237104.99(169119.94,296594.34)	5858.46(4180.60,7333.99)	165960.75(95503.39,232655.28)	1419.03(813.36,1991.48)	-4.70(-4.98,-4.42)
Tajikistan	48974.52(29643.07,68472.53)	6783.85(4084.21,9500.32)	76322.30(46781.20,106863.49)	5518.80(3437.11,7642.46)	-0.91(-1.31,-0.51)
Tanzania	151202.88(97365.62,208171.63)	5360.18(3442.99,7388.04)	285350.18(171930.82,416670.62)	4465.49(2647.04,6526.82)	-0.63(-0.71,-0.55)
Thailand	393778.91(227249.80,577668.72)	4295.03(2480.08,6288.56)	761170.33(437007.28,1131471.67)	2513.67(1444.13,3734.14)	-2.18(-2.40,-1.96)
The Bahamas	1468.21(845.47,2028.37)	3638.59(2103.48,5014.82)	2487.03(1466.76,3544.94)	2259.90(1331.52,3217.91)	-1.53(-1.61,-1.45)
The Gambia	6646.07(4363.37,9362.19)	7518.72(4975.99,10527.46)	19820.92(12889.92,27867.64)	8005.53(5206.87,11220.08)	0.14(0.01,0.27)
Timor-Leste	5570.97(3070.18,8202.73)	7949.08(4443.28,11570.90)	17699.47(11176.82,24780.78)	7777.96(4875.87,10930.95)	0.15(-0.03,0.33)
Togo	25548.09(16426.10,35081.69)	8220.18(5310.45,11224.55)	75187.09(48645.32,106484.66)	7865.40(5162.18,10995.58)	-0.15(-0.34,0.04)
Tokelau	24.21(11.10,38.19)	6566.43(2932.47,10482.82)	21.26(11.89,31.37)	5180.89(2889.30,7657.76)	-0.92(-1.05,-0.79)
Tonga	485.81(248.16,746.83)	3310.84(1660.86,5128.91)	737.49(442.67,1059.16)	3408.66(2036.17,4902.15)	0.26(0.12,0.40)
Trinidad and Tobago	11475.93(7712.36,14808.89)	5288.64(3486.19,6904.97)	15102.44(9727.52,21183.27)	2859.15(1833.45,4017.95)	-2.44(-2.70,-2.19)
Tunisia	50287.53(29338.16,73719.67)	4086.53(2360.83,6035.06)	92876.29(50785.59,144222.67)	2690.13(1467.59,4189.06)	-1.60(-1.70,-1.49)
Turkey	481667.25(307760.47,663657.29)	5559.70(3564.34,7632.56)	621402.14(399109.42,868221.25)	2552.48(1636.08,3567.55)	-2.70(-2.88,-2.53)
Turkmenistan	32413.40(20129.91,43065.91)	6309.40(3896.09,8395.41)	89077.71(57863.37,123191.86)	8215.48(5376.81,11257.15)	0.70(0.21,1.18)
Tuvalu	152.83(65.88,249.21)	8543.66(3592.89,13988.20)	201.89(113.77,290.31)	7230.22(4065.74,10436.52)	-0.58(-0.68,-0.49)
Uganda	121217.89(77342.10,170813.54)	7374.64(4721.35,10359.92)	163807.25(103646.28,236475.94)	4507.78(2875.17,6471.00)	-2.35(-2.68,-2.01)
Ukraine	1522606.99(1029245.05,1920523.84)	7890.43(5270.75,10008.59)	1000273.78(622095.17,1398066.14)	4655.03(2896.25,6518.81)	-2.32(-2.59,-2.05)
United Arab Emirates	6298.57(4245.40,8752.72)	6451.99(4399.11,8824.18)	21262.54(12966.11,30383.14)	3258.37(2030.21,4583.68)	-0.76(-1.23,-0.28)
United Kingdom	875155.90(676560.31,1022663.27)	3372.09(2599.76,3948.92)	345144.00(248525.50,434342.76)	893.89(645.50,1122.20)	-4.72(-4.97,-4.47)
United States	1652666.22(1208248.70,2024303.73)	1855.06(1351.71,2277.41)	1883441.82(1210416.11,2549495.67)	1142.28(734.10,1544.32)	-2.10(-2.41,-1.80)
Uruguay	49175.01(30665.90,65836.77)	4621.30(2869.52,6205.58)	33652.33(21138.30,44779.01)	2092.16(1324.44,2778.22)	-2.89(-3.10,-2.67)

Uzbekistan	171038.52(114453.84,224504.32)	5541.75(3717.20,7260.77)	319607.46(212722.85,426002.52)	4689.36(3040.00,6292.45)	-1.02(-1.43,-0.62)
Vanuatu	1719.92(1149.12,2389.82)	10831.71(7313.96,14893.39)	4078.42(2697.19,5482.90)	8921.75(5911.17,11967.22)	-0.79(-0.87,-0.72)
Venezuela	87395.33(64785.42,106569.47)	3502.72(2593.55,4273.81)	192461.00(120249.76,276423.13)	2400.71(1509.67,3432.93)	-1.88(-2.17,-1.59)
Vietnam	944784.71(564230.87,1371636.16)	9016.35(5399.01,13061.98)	2249691.81(1512102.59,3030405.79)	8745.05(5905.81,11687.36)	0.33(0.13,0.53)
Virgin Islands, U.S.	623.20(334.47,930.05)	2911.79(1551.81,4337.65)	655.76(379.86,976.03)	1320.94(750.36,1983.36)	-2.36(-2.51,-2.20)
Yemen	101025.86(52089.86,158246.90)	8270.49(4266.22,12898.64)	210334.93(115146.13,325997.20)	6104.32(3346.98,9417.96)	-1.24(-1.37,-1.11)
Zambia	45337.88(25418.65,67987.42)	6373.86(3609.78,9493.91)	113394.38(63130.26,171223.09)	6561.94(3609.56,9898.39)	-0.15(-0.30,-0.01)
Zimbabwe	49876.85(33856.81,67676.38)	5029.60(3412.44,6811.29)	137501.86(90407.24,195177.86)	7981.05(5278.83,11159.84)	2.21(1.53,2.89)
